# A LDH‐Derived Metal Sulfide Nanosheet‐Functionalized Bioactive Glass Scaffold for Vascularized Osteogenesis and Periprosthetic Infection Prevention/Treatment

**DOI:** 10.1002/advs.202403009

**Published:** 2024-08-19

**Authors:** Yixin Bian, Tingting Hu, Kexin Zhao, Xuejie Cai, Mengyang Li, Chaoliang Tan, Ruizheng Liang, Xisheng Weng

**Affiliations:** ^1^ Department of Orthopedic Surgery State Key Laboratory of Complex Severe and Rare Diseases Peking Union Medical College Hospital Chinese Academy of Medical Science and Peking Union Medical College Beijing 100730 P. R. China; ^2^ Department Electrical and Electronic Engineering The University of Hong Kong Pokfulam Road Hong Kong, SAR 999077 P. R. China; ^3^ State Key Laboratory of Chemical Resource Engineering Beijing Advanced Innovation Center for Soft Matter Science and Engineering Beijing University of Chemical Technology Beijing 100029 P. R. China; ^4^ Quzhou Institute for Innovation in Resource Chemical Engineering Quzhou 324000 P. R. China

**Keywords:** layered double hydroxides, periprosthetic infection, photothermal therapy, sulfide, vascularized osteogenesis

## Abstract

Periprosthetic infection and prosthetic loosing stand out as prevalent yet formidable complications following orthopedic implant surgeries. Synchronously addressing the two complications is long‐time challenging. Herein, a bioactive glass scaffold (BGS) functionalized with MgCuFe‐layered double hydroxide (LDH)‐derived sulfide nanosheets (BGS/MCFS) is developed for vascularized osteogenesis and periprosthetic infection prevention/treatment. Apart from the antibacterial cations inhibiting bacterial energy and material metabolism, the exceptional near‐infrared‐II (NIR‐II) photothermal performance empowers BGS/MCFS to eliminate periprosthetic infections, outperforming previously reported functionalized BGS. The rough surface topography and the presence of multi‐bioactive metal ions bestow BGS/MCFS with exceptional osteogenic and angiogenic properties, with 8.5‐fold and 2.3‐fold enhancement in bone mass and neovascularization compared with BGS. Transcriptome sequencing highlights the involvement of the TGF‐β signaling pathway in these processes, while single‐cell sequencing reveals a significant increase in osteoblasts and endothelial cells around BGS/MCFS compared to BGS.

## Graphical abstract

A bioactive glass scaffold functionalized with LDH‐derived sulfide nanosheets is proposed for bioactive ion‐mediated vascularized osteogenesis and photothermal effect‐mediated antibacterial, synchronously addressing periprosthetic infection and prosthesis loosening.

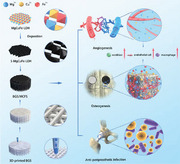

## 1 Introduction

Orthopedic implants encompassing total knee/hip prosthesis, fixation instruments, and bone defect scaffolds are commonly applied in clinical practice to treat amounts bone diseases including osteoarthritis, osteonecrosis of the femoral head, fractures, critical‐size bone defects, etc.^[^ ^1^, ^2^, ^3^, ^4^, ^5^ ^]^ However, the implants can be frequently compromised by postoperative periprosthetic infection and prosthetic loosening, ultimately leading to prosthesis failure.^[^ ^6^, ^7^, ^8^, ^9^ ^]^ Periprosthetic infection occurs primarily due to suboptimal aseptic surgical procedures and the lack of antibacterial capabilities of the prosthesis,^[^ ^10^, ^11^, ^12^ ^]^ while inadequate osteogenesis and osteointegration properties of the implants contribute to prosthetic loosening.^[^ ^13^, ^14^ ^]^ To date, several scaffolds such as magnesium (Mg) alloy, chitosan, hydroxyapatite (HAp), and bioactive glass scaffolds (BGS) have been designed for advanced orthopedic prosthesis, of which BGS with excellent biocompatibility, favorable biomechanical characteristics, and intrinsic osteogenic properties attracts amounts attention.^[^ ^15^, ^16^ ^]^ However, the osteogenic and angiogenic capabilities of pristine BGS are yet unsatisfactory and have failed to reconstruct critical‐sized bone defects. Thus, several strategies have been proposed to improve the vascularized bone reconstruction capability of BGS. For instance, Eriksson et al.^[^ ^17^ ^]^ developed an amorphous BGS with a porous interior structure that is in favor of bone and vessel ingrowth. Niu et al.^[^ ^18^ ^]^ designed a bone morphogenetic protein 2 (BMP‐2) sustained‐release system based on hierarchically mesoporous BGS for enhanced osteogenesis and orchestrated scaffold degradation. Nevertheless, relying solely on surficial or internal structural modifications is insufficient to achieve ideal bone reconstruction, while incorporating bioactive factors into scaffold raises biosafety concerns such as oncogenesis and ectopic ossification.^[^ ^19^, ^20^, ^21^ ^]^ In addition, the occurrence of periprosthetic infection not only significantly inhibits normal bone regeneration and remodeling, but also is a catastrophic complication of prosthesis implantation. However, modifying BGS to empower it with antibacterial abilities while maintaining or enhancing its osteogenic properties is especially a persistent difficulty.^[^ ^10^, ^22^ ^]^ Recently, efforts have been made to design bifunctional BGS‐based scaffolds that can synergistically inhibit bacteria and promote bone regeneration. For example, metallic silver nanoparticles were embedded in mesoporous BGS to endow it with additional antibacterial abilities, while the internal mesoporous structure contributed to its enhanced osteogenic properties.^[^ ^23^ ^]^ In another study, single‐atomic iron functionalized BGS could generate photothermal‐deriving hyperthermia to eliminate bacteria and promote bone regeneration.^[^ ^24^ ^]^ However, these scaffolds could not eliminate bacteria to thoroughly treat periprosthetic infection due to the insufficient antibacterial abilities of simple metal ions or the limited penetration capabilities of the involved first near‐infrared (NIR‐I) region light, and their vascularized osteogenic properties remain suboptimal. Thus, the development of a versatile scaffold that amalgamates potent antibacterial and vascularized osteogenesis properties to simultaneously address periprosthetic infection and prosthetic loosening can offer significant clinical benefits for patients receiving orthopedic implants.

Photothermal therapy (PTT), as a promising therapeutic strategy for anti‐bacterial and anti‐tumor treatments, has sparked increasing interest due to its exceptional benefits of high selectivity, good specificity, non‐invasiveness, and minimal side effects.^[^ ^25^, ^26^, ^27^, ^28^ ^]^ PTT employs photothermal agents to convert light energy into localized hyperthermia under NIR laser irradiation, inducing cell membrane rupture, protein/enzyme denaturation, and bacterial or cellular structure damage.^[^ ^29^, ^30^, ^31^, ^32^ ^]^ As nanotechnology swiftly progresses, various inorganic NIR‐II (maximum permissible exposure and higher penetration depth than NIR‐I) photothermal agents have been developed for PTT antibacterial because of their excellent chemical stability, low phototoxicity, adjustable composition and structure, such as noble metals, transition metal chalcogenides (TMCs), and MXenes.^[^ ^33^, ^34^, ^35^, ^36^, ^37^ ^]^ Among them, ultrathin TMC nanosheets with quantum confinement effect, favorable electronic band structure, and predominant optical absorption have been found to be promising candidates for PTT.^[^ ^38^, ^39^ ^]^ To prepare ultrathin TMC nanosheets, several methods (e.g., wet‐chemical synthesis, chemical vapor deposition, mechanical exfoliation, etc.) have been proposed.^[^ ^40^, ^41^ ^]^ Particularly, the topological transformation of layered double hydroxide (LDH) nanosheets into TMC nanosheets has been proven to be a simple and powerful strategy,^[^ ^42^, ^43^ ^]^ which can maintain the sheet‐like structure and metal composition of LDH, and endow TMC nanosheets with uniform morphology and excellent photothermal conversion properties. For example, CuFe_2_S_3_ and CoFeMn dichalcogenide nanosheets prepared by hydrothermal sulfurization of CuFe‐LDH and CoFeMn‐LDH precursors exhibited remarkable PTT efficacy with photothermal conversion efficiency (η) of ≈55.86% and 89.0%, respectively,^[^ ^44^, ^45^ ^]^ effectively inducing cancer cells death and tumor elimination under NIR irradiation. In addition, LDH‐based materials have been reported to possess osteogenesis and angiogenesis properties owing to the existence of bioactive Mg^2+^, calcium (Ca^2+^), copper (Cu^2+^), iron (Fe^3+^), europium (Eu^3+^), and strontium (Sr^2+^) ions.^[^ ^46^, ^47^, ^48^, ^49^, ^50^, ^51^, ^52^ ^]^ For instance, MgAlEu‐LDH nanosheet‐functionalized porous HAp exhibited favorable vascularized osteogenesis performance.^[^ ^53^ ^]^ Therefore, regulating the composition of LDH followed by topological transformation is expected to construct versatile LDH‐derived polymetallic sulfide nanosheets with osteogenic and photothermal properties for synergistic bone repair and NIR‐II antibacterial therapy.

**Figure 1:**
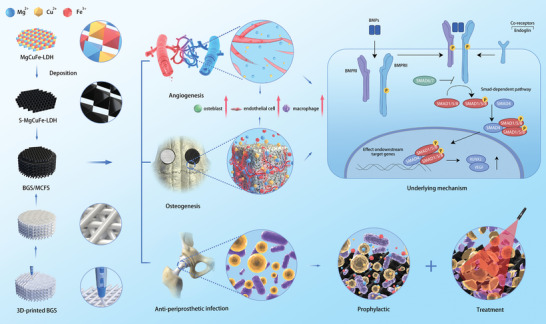
Schematic illustration of the fabrication of a versatile MCFS nanosheet‐functionalized 3D‐printed BGS for periprosthetic infection prevention/treatment and vascularized osteogenesis.

In this study, we develop a bifunctional MgCuFe‐LDH‐derived polymetallic sulfide (MCFS) nanosheets incorporating bioactive Mg^2+^, Cu^2+^, and Fe^3+^ ions via a facile sulfurization process based on topological transformation effect. By integrating MCFS into a 3D‐printed BGS, the BGS/MCFS with prophylactic/photothermal therapeutic capabilities against periprosthetic infections and remarkable vascularized osteogenic properties is fabricated (**Figure** **1**). Specifically, the MCFS containing polymetallic ions demonstrates potent antibacterial effects, notably inhibiting the proliferation of *Methicillin‐resistant Staphylococcus aureus* (*MRSA*, a drug‐resistant bacteria) by over 80%. The RNA sequence of *MRSA* shows that the basic energy production and nutrient metabolism of *MRSA* co‐cultured with MCFS were obviously inhibited. More importantly, the exceptional NIR‐II photothermal conversion efficiency of MCFS facilitates the scaffold temperature increase to ≈70 °C under 1 W cm^−2^ light irradiation, which is verified to effectively eradicate bacteria (with an efficiency of 100%) around implants and prevent subsequent osteomyelitis in a rabbit periprosthetic infection model. The bacterial eradication capabilities highlight the superiority of BGS/MCFS compared to previously reported functionalized BGS (Table S1, Supporting Information). Furthermore, the incorporation of MCFS into the BGS leads to increased surface roughness and enhanced cellular adhesion. The sustained release of Mg^2+^, Cu^2+^, and Fe^3+^ from MCFS endows the scaffold with excellent osteogenic and angiogenic properties. In a rabbit critical‐size cranial defect model, the BGS/MCFS induces an impressive 8.5‐fold increment in regenerated bone mass and a 2.3‐fold increment in neovascularization compared with pristine BGS, which is remarkably preponderant than other previously reported modified BGS (Table S2, Supporting Information). Transcriptome sequencing and subsequent molecular verification reveal that the TGF‐β signal pathway contributes to the vascularized osteogenic properties of BGS/MCFS. Single cell sequencing further demonstrates a remarkable increment in osteoblasts and endothelial cells around BGS/MCFS compared with pristine BGS. In summary, our study introduces a bifunctional scaffold characterized by prophylactic and therapeutic efficiency against periprosthetic infections, as well as exceptional osteogenic and angiogenic properties, which are promising in synchronously addressing periprosthetic infection and prosthetic loosening, thus benefiting patients requiring prosthesis implantation.

## 2 Results and Discussion

**Figure 2:**
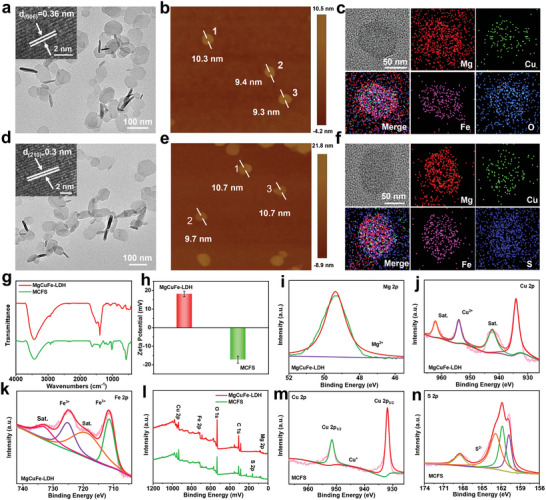
Characterizations of MgCuFe‐LDH and MCFS NSs. a) TEM, b) AFM, and c) EDX elemental mapping images of MgCuFe‐LDH. d) TEM, e) AFM, and f) EDX elemental mapping images of MCFS NSs. g) FT‐IR spectra and h) Zeta potentials of MgCuFe‐LDH and MCFS NSs. i) Mg 2p, j) Cu 2p and k) Fe 2p XPS spectra of MgCuFe‐LDH. l) Wide XPS spectra of MgCuFe‐LDH and MCFS NSs. m) Cu 2p and n) S 2p XPS spectra of MCFS NSs.

The MCFS NSs were prepared via a facile two‐step process.^[^ ^44^, ^45^ ^]^ Firstly, a bottom‐up synthesis method was utilized to prepare MgCuFe‐LDH NSs as precursors. Secondly, MgCuFe‐LDH NSs were transformed into MCFS NSs through topological transformation using thioacetamide (TAA) as the sulfur source. X‐ray diffraction (XRD) was performed to investigate the transformation from MgCuFe‐LDH to MCFS NSs. As shown in Figure S1a (Supporting Information), typical peaks at 11.55° and 23.01° are in accordance with (003) and (006) planes of MgCuFe‐LDH. After sulfurization, the XRD pattern of MCFS NSs matches well with the Cu_9_S_8_ phase (JCPDS 36–0379), Cu_5_FeS_4_ phase (JCPDS 25–1424), and CuFe_2_S_3_ phase (JCPDS 47–1749), demonstrating the successful preparation of mixed metal sulfide nanosheets (Figure S1b, Supporting Information). It should be noted that Mg was doped in the MCFS NSs without forming sulfide. Transmission electron microscopy (TEM) image shows the nanosheet‐like morphology of MgCuFe‐LDH sample with a size of 60–100 nm (**Figure** **2**a). Continuous lattice fringes with an interplanar spacing of ≈0.36 nm observed from high‐resolution TEM (HRTEM) image (Figure 2a) are assigned to the (006) plane of LDH crystal. The thickness of MgCuFe‐LDH NSs characterized by atomic force microscopy (AFM) is 9.3–10.3 nm (Figure 2b; Figure S2a, Supporting Information). The energy‐dispersive X‐ray (EDX) elemental mapping reveals the homogeneous distribution of Mg, Cu, Fe, and O elements on LDH NSs (Figure 2c). Importantly, the MCFS NSs maintain the nanosheet‐like morphology of MgCuFe‐LDH NSs with unaltered size and slightly increased thickness of 9.7–10.7 nm (Figure 2d,e; FIgure S2b, Supporting Information). The measured lattice fringe of ≈0.30 nm matches well with the (210) plane of CuFe_2_S_3_ phase (Figure 2d). Moreover, a uniform dispersion of Mg, Cu, Fe, and S is observed from MCFS NSs according to EDX elemental mapping images (Figure 2f), implying a successful sulfurization reaction and the presence of doped Mg ions.

Fourier transform infrared spectroscopy (FT‐IR) was conducted to further characterize the structural transformation from MgCuFe‐LDH to MCFS NSs. In Figure 2g, the band at 1384 cm^−1^ (N─O stretching vibration in NO_3_^−^) was observed in MgCuFe‐LDH NSs, while it almost disappeared in MCFS NSs, implying the replacement of M─OH by M─S (M═Cu, Fe). Then, we investigated the physicochemical properties of MgCuFe‐LDH and MCFS NSs. As shown in Figure 2h, MgCuFe‐LDH NSs display a positive potential of ≈18.2 ± 1.4 mV in water, which changes to ≈−17.3 ± 1.9 mV after sulfurization with black color (Figure S3, Supporting Information). Significant Tyndall effect of MgCuFe‐LDH and MCFS NSs in water was observed from day 0 to day 7, implying their excellent dispersibility and storage stability (Figure S4, Supporting Information). The chemical composition and valence states of MgCuFe‐LDH and MCFS NSs were characterized by X‐ray photoelectron spectroscopy (XPS). For the MgCuFe‐LDH precursor (Figure 2i–l), peak at 49.4 eV in Mg 2p spectrum is assignable to Mg^2+^. Peaks at 953.9 and 934.1 eV in Cu 2p spectrum with satellite peaks at 962.1 and 942.4 eV correspond to Cu^2+^ 2p_1/2_ and 2p_3/2_, respectively. In terms of Fe 2p XPS spectrum, peaks at 725.3 and 711.6 eV with satellite peaks at 733.4 and 719.4 eV correspond to Fe^3+^ 2p_1/2_ and 2p_3/2_, respectively. After sulfurization, the valence state of Mg is still Mg^2+^ (Figure S5a, Supporting Information). The characteristic peaks of Cu^+^ 2p_1/2_ and 2p_3/2_ at 951.7 and 931.8 eV were observed (Figure 2m). The Fe 2p_3/2_ peak at 707.9 eV and Fe 2p_1/2_ band at 721.2 eV also appeared (Figure S5b, Supporting Information), which are assigned to Fe^2+^. The relative ratio of Fe^2+^ and Fe^3+^ is calculated to be 1:2.73. Moreover, in XPS S 2p peaks spectrum, peaks at 168.5, 162.9, and 161.8 eV correspond to S^2−^ (Figure 2n). The above results indicated that sulfurization could induce partial reduction of Cu^2+^ and Fe^3+^ to Cu^+^ and Fe^2+^.

**Figure 3:**
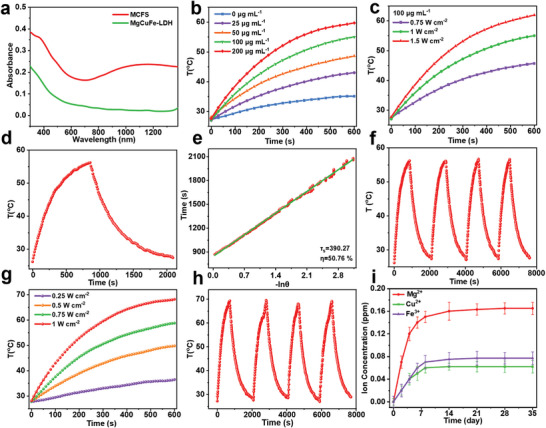
Photothermal performance of MCFS. a) UV–vis‐NIR absorbance spectra of MgCuFe‐LDH and MCFS NSs. b) Temperature curves of MCFS suspensions at different concentrations (0, 25, 50, 100, and 200 µg mL^−1^) upon 1064 nm laser irradiation (1.0 W cm^−2^). c) Temperature curves of 100 µg mL^−1^ MCFS suspensions upon 1064 nm laser irradiation with different laser power densities (0.75, 1, and 1.5 W cm^−2^). d) Temperature rise and fall curve upon 1064 nm laser irradiation. e) Time versus negative natural logarithm of the driving force temperature (τ_s_ = 390.27 s) from the cooling test. f) Thermal stability tests of MCFS. g) Temperature curves of BGS/MCFS scaffolds upon 1064 nm laser irradiation with different laser power densities (0.25, 0.5, 0.75, and 1 W cm^−2^). h) Photostability tests of BGS/MCFS. i) Mg^2+^, Cu^2+^, and Fe^3+^ release from BGS/MCFS.

As displayed in UV–vis–NIR absorption spectra (**Figure** **3**a), MgCuFe‐LDH NSs exhibited weak absorption intensity in NIR region, while after sulfurization, the absorbance in NIR region obviously increased. Based on this, the temperature change of different samples upon 1064 nm laser irradiation was monitored. Under 1 W cm^−2^ laser irradiation, the temperature elevation (*Δ*T) ranging from 7.8 to 32.1 °C was observed with the rise of MCFS concentration (0–200 µg mL^−1^, Figure 3b). The apparent visual changes in temperature were also monitored by a thermal infrared imaging device (Figure S6, Supporting Information). When the concentration of MCFS was 100 µg mL^−1^, the *Δ*T increased from 18.3 to 34.2 °C by enhancing the laser power (0.75–1.5 W cm^−2^, Figure 3c). These results suggested that the photothermal performance of MCFS NSs was positively correlated with concentration and laser power. According to the fitting‐cooling curve, the photothermal conversion efficiency (η) of MCFS NSs was determined to be 50.76% (Figure 3d,e), verifying the satisfactory photothermal performance of MCFS NSs, which can be attributed to the presence of Cu_9_S_8_, Cu_5_FeS_4_, and CuFe_2_S_3_ that are reported high‐performance photothermal reagents.^[^ ^39^, ^44^ ^]^ Additionally, photothermal stability tests of MCFS NSs were conducted. After four heating‐cooling cycles, there was no significant deterioration in photothermal conversion performance (Figure 3f), indicating its stable photothermal conversion capability.

Based on the above results, the BGS was placed in MCFS NSs suspension (1 mg mL^−1^) to prepare BGS/MCFS composite scaffolds. The digital photos showed that the surface of the BGS/MCFS scaffold was black (Figure S7, Supporting Information), suggesting that the BGS (white) was completely covered by MCFS NSs. Scanning electron microscopy (SEM) was used to investigate their surface micro‐morphologies. As shown in Figure S7 (Supporting Information), the surface of BGS had a relatively smooth structure, while the surface of BGS/MCFS was much rougher, which is beneficial for cell adhesion. The EDS elemental mapping images indicated the uniform distribution of Si, P, O, Ca (from BGS), Mg, Cu, Fe and S (MCFS NSs) elements on BGS/MCFS (Figure S8, Supporting Information). The photothermal properties of BGS/MCFS were further studied. It was found that *Δ*T of BGS/MCFS could be tuned from 8.7 to 40 °C by enhancing the laser power density (0.25–1.0 W cm^−2^, Figure 3g; Figure S9, Supporting Information), and the repeatable and regenerated photothermal performance of BGS/MCFS was also verified by photothermal stability tests (Figure 3h). Besides, the release of Mg^2+^, Cu^2+^ and Fe^3+^ from BGS/MCFS was characterized by inductively coupled plasma‐atomic emission spectroscopy (ICP‐AES). As displayed in Figure 3i, BGS/MCFS could sustainably release aforementioned bioactive ions, which are beneficial to bone regeneration and bacterial inhibition.

**Figure 4:**
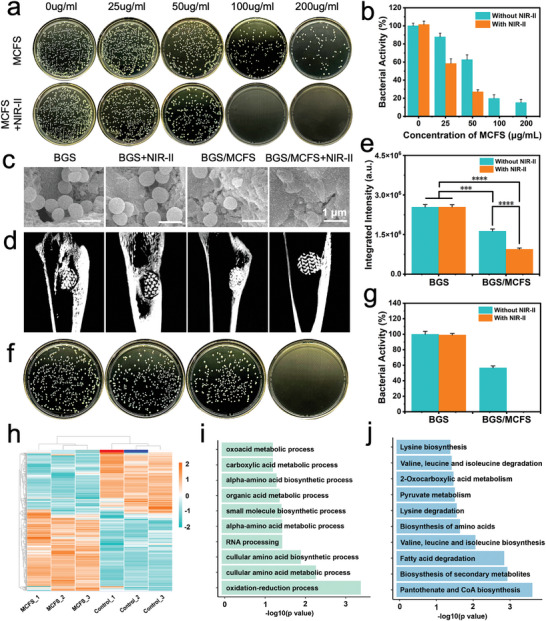
Periprosthetic infection prevention and treatment capability of BGS/MCFS. a) Digital photographs and b) quantitative profile of the bacterial colony formed by remaining *MRSA* after incubation with different concentration MgCuFe‐LDH suspension with or without 1064 nm laser irradiation. c) SEM images of *MRSA* inoculated on BGS or BGS/MCFS with or without 1064 nm laser irradiation. d) Micro‐CT images of rabbit tibia modeled with periprosthetic infection and treated with indicated therapies. e) Quantitative analysis of the integrated intensity of sagittal Micro‐CT images evaluating the severity of osteomyelitis. f) Digital photographs and g) corresponding counting of colonies incubated from the homogenated muscle tissue around BGS or BGS/MCFS of different therapy groups. h) Heatmap illustrating the differentially expressed genes between MCFS group and control group. i) GO and j) KEGG analysis enriching the downregulated genes in different biological processes and signal pathways (showing Top 10). Data are expressed as mean ± standard deviation (S.D.) (*n* = 3). Statistical comparisons were made by one‐way ANOVA (for multiple comparisons): ^***^ *p* < 0.001, ^****^ *p* < 0.0001.

To evaluate the in vitro drug‐resistant bacteria elimination performance of MCFS NSs and BGS/MCFS, *MRSA* were co‐incubated with various concentrations of MCFS NSs (0, 25, 50, 100, and 200 µg mL^−1^) with or without 1064 nm laser irradiation followed by inoculation in agar plates for 12 h propagation. The results indicated the bacterial colonies were significantly decreased with the MCFS NSs concentration increased, which reduced to 19.8% and 15.4% of that in control group upon 100 and 200 µg mL^−1^ MCFS NSs, respectively (**Figure** **4**a,b). More importantly, complete bacterial eradication was observed in MCFS + NIR‐II groups with 100 and 200 µg mL^−1^ MCFS NSs, demonstrating the potent antibacterial capabilities generated by the prominent photothermal effects of MCFS NSs (Figure 4a,b). In addition, to verify the unselective antibacterial performance of MCFS NSs, *Escherichia coli* (*E. coli*, Gram‐negative bacteria) and *Staphylococcus aureus* (*S. aureus*, Gram‐positive bacteria) were employed. As shown in Figure S10 (Supporting Information), significantly inhibited *E. coli* proliferation was observed with increased MCFS NSs concentration and complete elimination of *E. coli* was obtained in 100 and 200 µg mL^−1^ MCFS NSs groups under NIR‐II irradiation. Similar results also reappeared in colony culture experiment for *S. aureus* (Figure S11, Supporting Information), confirming the comprehensive antibacterial performance of MCFS NSs. Subsequently, to more intuitively verify the antibacterial efficacy of the BGS/MCFS, *MRSA*, *E. coli*, and *S. aureus* were directly seeded on the surface of BGS/MCFS followed by 1064 nm laser irradiation (0 or 1 W cm^−2^) for 10 min. The SEM images demonstrated the bacteria in BGS, BGS + NIR‐II exhibited extensive adhesion and uniform spherical form while bacteria in BGS/MCFS group presented reduced number and shrink appearance (Figure 4c; Figures S12 and S13, Supporting Information), which indicates the bacterial inhibition capability of BGS/MCFS. Furthermore, bacteria in BGS/MCFS + NIR‐II lost primitive shape and bore a shriveled or fragmentized morphology (Figure 4c; Figures S12 and S13, Supporting Information), highlighting the potent bacteria extermination ability of BGS/MCFS under NIR‐II laser irradiation.

Encouraged by the excellent in vitro antibacterial performance, the in vivo anti‐periprosthetic infection of BGS/MCFS was explored. The bacterial‐adhered BGS or BGS/MCFS were implanted into the penetrating bone defect on the upper ending of the tibia of New Zealand white rabbits to simulate the postoperative periprosthetic infection in clinic. One day after surgery, the scaffolds in BGS+NIR‐II and BGS/MCFS+ NIR‐II groups were irradiated with a 1064 nm laser (0 or 1 W cm^−2^) through tibial skin. The corresponding tibial specimen was harvested, fixed, and subjected to Micro‐computed tomography (Micro‐CT) imaging and reconstruction 1 month after scaffold implantation. Obvious osteomyelitis of the tibia was developed in BGS and BGS + NIR‐II groups, indicated by diffused abnormal high signal intensity around the bone defects and bone marrow cavity, while regular and clear Micro‐CT images were observed in the BGS/MCFS + NIR‐II group (Figure 4d,e). Of note, BGS/MCFS without NIR‐II irradiation also partially inhibit the bacterial amplification and retard osteomyelitis, with a 57% reduction in abnormal signal intensity (Figure 4d,e), which are considered to attributed to the sustained released antibacterial metal ions including Mg^2+^, Cu^2+^, Fe^3+^. We further histologically accessed the bone regeneration outcomes of BGS, BGS + NIR‐II, BGS/MCFS, and BGS/MCFS + NIR‐II treatments in infected rabbit proximal tibial periprosthetic models using hematoxylin‐eosin (H&E) staining. As expected, significantly eroded bone structure and diffused neutrophil infiltration were observed in the *MRSA*‐infected BGS, BGS + NIR‐II, and BGS/MCFS groups due to uncontrolled infection, while normal bone structure with favorable osteogenesis and osteointegration were found in the BGS/MCFS + NIR‐II group (Figure S14, Supporting Information). Furthermore, to eliminate the adverse impact of uncontrolled periprosthetic infection on bone regeneration in BGS, BGS + NIR‐II, and BGS/MCFS groups, a parallel control series using a non‐infected rabbit proximal tibial bone defect model was employed for comparison. As illustrated in Figure S14 (Supporting Information), in the non‐*MRSA* series, the BGS/MCFS and BGS/MCFS + NIR‐II groups demonstrated remarkable but similar enhanced osteogenesis and bone reconstruction compared with the BGS and BGS + NIR‐II groups. More importantly, bone regeneration in the *MRSA*‐infected BGS/MCFS + NIR‐II group was comparable to that in the non‐infected BGS/MCFS and BGS/MCFS + NIR‐II groups (Figure S14, Supporting Information). These findings verify that the additional NIR‐II irradiation poses no adverse impact on the osteogenic efficacy of BGS/MCFS while effectively eliminating periprosthetic infection. In addition, an identical mass muscle tissue around the bacterial‐adhered scaffold was harvested and ground for the following LB agar inoculation. As depicted in Figure 4f, the highest number of colony formations was observed in the BGS and BGS + NIR‐II groups and fewer colonies were formed in the BGS/MCFS group, while no colony formation was observed in the BGS/MCFS + NIR‐II group. The corresponding results were further confirmed by colony counting using Image J, as illustrated in Figure 4g. The in vivo bacterial eradication capabilities highlight the superiority of BGS/MCFS compared to previously reported modified BGS in treating periprosthetic infection (Table S1, Supporting Information).

To elucidate the underlying mechanism of the intrinsic antibacterial properties of MCFS NSs, the RNA sequence for *MRSA* co‐cultured with normal medium or medium with MCFS NSs (100 µg mL^−1^) was conducted. The volcano map (Figure S15, Supporting Information) and heatmap (Figure 4h) illustrate the differentially expressed genes (P < 0.05&|log2 Fold Change| >1), where a total of 988 differentially expressed genes were identified, including 549 upregulated genes (marked in red) and 439 downregulated genes (marked in green). Gene Ontology (GO) analysis was subsequently involved to conduct the downregulated gene enrichment. As shown in Figure 4i, the downregulated genes were enriched into various critical physiological processes that are essential for the basic life activities of bacteria, including oxidation‐reduction reaction, amino acid metabolism, and RNA processing, etc. Of note, oxidation‐reduction processes are vital for bacterial energy production, nutrient metabolism, and defense against oxidative stress. Suppressing oxidation‐reduction processes can directly reduce the energy production and nutrient metabolism efficiency of bacteria, thus significantly undermining bacterial vitality.^[^ ^54^ ^]^ The Kyoto Encyclopedia of Genes and Genomes (KEGG) analysis was further employed and revealed the inhibition of the pantothenate and coenzyme A synthesis, secondary metabolic, and multiple amino acids by MCFS NSs compared with the control group (Figure 4j). Importantly, inhibiting pantothenate and CoA biosynthesis leads to CoA deficiency, disrupting fatty acid metabolism, impairing energy production, and compromising cell membrane integrity, ultimately causing bacterial cell death.^[^ ^55^ ^]^ These findings suggest that the antibacterial mechanism of MCFS primarily involves direct interference with bacterial energy production and disruption of nutrient metabolism.

**Figure 5:**
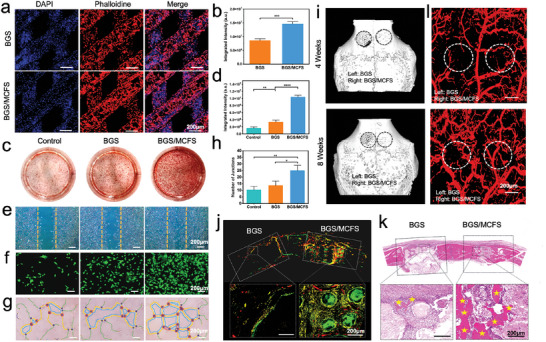
Vascularized osteogenesis properties of BGS/MCFS. a) CLSM images of hBMSCs stained with DAPI (displaying blue fluorescence) and rhodamine phalloidin (displaying red fluorescence) on the surface of BGS and BGS/MCFS after 1 day of incubation. b) Quantitative analysis of the fluorescence intensity of hBMSCs adhered on BGS and BGS/MCFS using ImageJ 1.52v software. c) Digital images of Alizarin red S staining of hBMSCs cultured with BGS and BGS/MCFS. d) Quantitative analysis of Alizarin red S staining images using ImageJ 1.52v software. e) Optical microscope images of scratch assay at 24 h accessing the migration activity of HUVECs cultured with BGS or BGS/MCFS. f) Optical microscope images of migrated HUVECs stained with Calcein‐AM in Transwell assay in control, BGS, and BGS/MCFS groups. g) Analytical images of tube formation assay co‐culturing HUVECs with BGS or BGS/MCFS on Matrigel. h) Quantitative analysis of the number of joint in tube formation experiments using ImageJ 1.52v software. i) Front view of 3D reconstructed Micro‐CT images indicating bone regeneration induced by BGS or BGS/MCFS 4 and 8 weeks after scaffold implantation. j) CLSM images of newly‐formed bone tissue labeled with Calcein‐AM and alizarin red around BGS or BGS/MCFS. k) H&E staining of regenerated bone induced by BGS or BGS/MCFS. l) 3D reconstructed Micro‐CT images of regenerated vessels around BGS or BGS/MCFS. Data are expressed as mean ± standard deviation (S.D.) (n = 3). Statistical comparisons were made by one‐way ANOVA (for multiple comparisons): ^*^ *p* < 0.05, ^**^ *p* < 0.01, ^***^ *p* < 0.001, ^****^ *p* < 0.0001.

To evaluate the osteogenic and angiogenic properties of BGS/MCFS, Cell counting kit‐8 (CCK‐8) assays were first conducted to explore the biocompatibility of prepared BGS/MCFS on days 1, 3, 5, and 7. The results illustrated that BGS/MCFS possesses excellent biocompatibility with comparable CCK‐8 values in the control, BGS, and BGS/MCFS group for both human bone marrow mesenchymal stem cells (hBMSCs) (Figure S16a, Supporting Information) and human umbilical vein endothelial cells (HUVECs) (Figure S16b, Supporting Information) were observed. Notably, the CCK‐8 values were slightly higher in BGS/MCFS group on day 7 compared with control and BGS groups, suggesting the proliferation‐promoting effects of BGS/MCFS for hBMSCs and HUVECs (Figure S16, Supporting Information). To evaluate the cell adhesion properties of BGS/MCFS, the hBMSCs were seeded on BGS or BGS/MCFS and observed under a confocal microscope after fluorescent staining. It turned out the hBMSCs can adhere and grow uniformly and densely on both BGS and BGS/MCFS, as revealed by confocal laser scanning microscopy (CLSM) images (**Figure** **5**a), while further quantitative analysis reveals significantly higher fluorescence intensity (1.7‐fold) in BGS/MCFS compared with BGS (Figure 5b), indicating the enhanced cellular adhesion properties of BGS/MCFS. SEM images further show remarkably more outstretched protrusion of hBMSCs adhered on BGS/MCFS surface, implying a more active locomotion and differentiation of hBMSCs in BGS/MCFS compared with BGS (Figure S17, Supporting Information).

Subsequently, the effects of BGS/MCFS on the osteogenic differentiation of hBMSCs were exploited by alizarin red S staining and alkaline phosphatase staining assays. Fourteen days after co‐culture, significantly more calcium depositions were observed in BGS/MCFS group compared with control and BGS groups (Figure 5c; Figure S18, Supporting Information). Quantitative analysis demonstrated 6.4 and 3.1‐fold of the OD value of Alizarin red S increment in BGS/MCFS group compared with control and BGS groups, respectively (Figure 5d). Similarly, extensive and deeper colored alkaline phosphatase was detected in BGS/MCFS group (Figure S19a, Supporting Information), with 7.5 and 3.5‐fold signal intensity compared with that in control and BGS groups (Figure S19b, Supporting Information), which verified the outstanding in vitro osteogenic capabilities of BGS/MCFS. Vascularization is a crucial fundamental precondition for subsequent qualified osteogenesis. To investigate the in vitro angiogenic properties of BGS/MCFS, scratch assay and transwell assay evaluating cell migration capacity were performed using HUVECs. As shown in Figure S20 (Supporting Information) and Figure 5e, a significantly enhanced cell migration ratio (3.4‐fold) indicated by thinner remaining scratch was found in the BGS/MCFS group compared with control group after 24 h healing. Similarly, the HUVECs passing through the transwell membrane were also remarkably increased by 5.1‐fold when co‐cultured with BGS/MCFS compared with that in control group (Figure 5f; Figure S21, Supporting Information). Of note, the scratch assay and transwell assay revealed BGS can induce HUVECs migration as well despite the induction‐strength being obviously weaker than that of BGS/MCFS (Figure 5e,f; Figure S21, Supporting Information). Furthermore, to verify the vascularity generation ability of BGS/MCFS more directly, tube formation assays were performed by incubating HUVECs on Matrigel. As presented in Figure S22 and Figure 5g, significantly more tube structures were seen in BGS/MCFS group compared with control and BGS groups. Further quantitative analysis revealed 2.4‐fold and 2.0‐fold increments of junction number (Figure 5h) and total length (Figure S23, Supporting Information) in BGS/MCFS group compared with control group. Interestingly, BGS that can enhance cell migration did not promote tube formation, demonstrating its inability to induce coordinately integration and assemble of migrated HUVECs as BGS/MCFS did.

To further access the vascularized osteogenesis properties of BGS/MCFS in vivo, critical‐sized calvaria defect models were established in New Zealand White Rabbits and implanted with BGS or BGS/MCFS. Four and eight weeks after scaffold implantation, the skulls were harvested and scanned by 3D reconstructed Micro‐CT. As shown in Figure 5i and Figure S24 (Supporting Information), significantly more new bone formation was found in BGS/MCFS group characterized by extended defect coverage and better osseointegration in both 4 and 8 weeks. Further quantitative morphometric analysis of Micro‐CT images indicated 2.6‐fold and 3.6‐fold bone volume (Figure S25a, Supporting Information), 2.1‐fold and 2.4‐fold bone mineral density (Figure S25b, Supporting Information), and 5.6‐fold and 8.5‐fold total bone mass (Figure S25c, Supporting Information) increment in BGS/MCFS group compared with BGS group at 4 and 8 weeks after scaffold implantation, respectively. The fold‐increment in bone formation was found to be significantly preponderant than those reported in previous studies involving other modification strategies for BGS (Table S2, Supporting Information). Moreover, fluorescent dyes including Calcein acetoxymethyl ester (Calcein‐AM) and Alizarin red S were intraperitoneally injected into modeled rabbits 4 and 6 weeks after scaffold implantation to identify new calcium deposition. Significantly enhanced and extensive fluorescence intensity was observed around BGS/MCFS while only sparse and scattered fluorescence could be detected around pristine BGS (Figure 5j). Similar results were also confirmed by the H&E (Figure 5k) and toluidine blue staining (Figure S26, Supporting Information), where remarkably more staggered distribution of new bone formation filling the space across the scaffold was observed in BGS/MCFS group but only marginal regenerated bone tissue could be detected in BGS group, demonstrating the superior osteogenic properties of BGS/MCFS compared with BGS.

The in vivo angiogenic properties of BGS/MCFS were evaluated by perfusing MICROFIL to fill microvascular bed of the skull and surrounding tissue for subsequent Micro‐CT scanning and reconstruction. The corresponding Micro‐CT images exhibited denser and multi‐branched neo‐vascular networks encircling the BGS/MCFS but only sparse newly‐formed vessels around BGS (Figure 5l). Further quantitative analysis demonstrated a 2.1‐fold and 2.3‐fold increment of vascular intensity in BGS/MCFS group compared with BGS group (Figure S27, Supporting Information) 4 and 8 weeks after implantation, respectively, demonstrating the excellent angiogenesis capabilities of BGS/MCFS.

**Figure 6:**
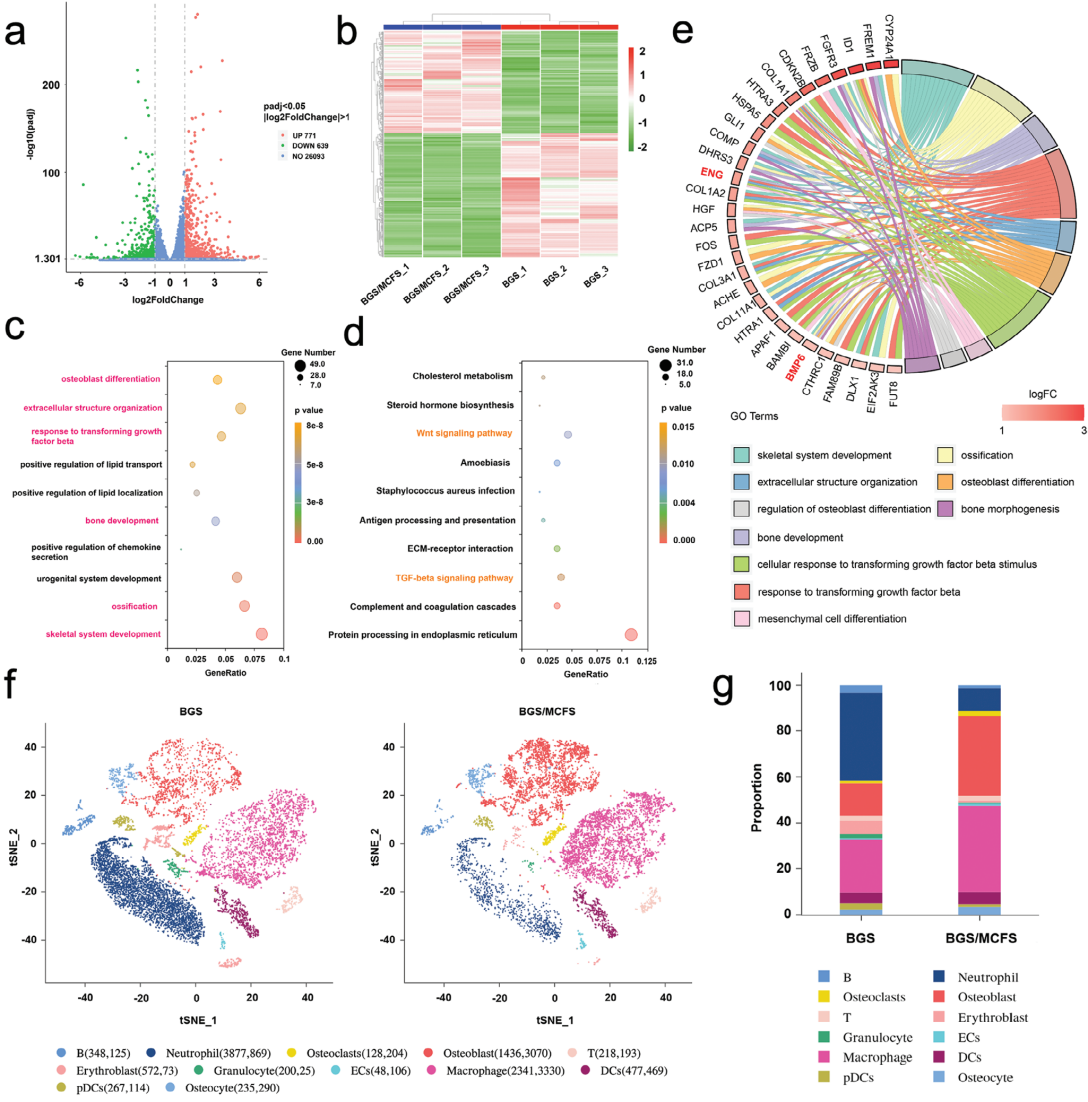
Underlying mechanism of the vascularized osteogenesis properties of BGS/MCFS. a) Volcano plot and b) heatmap showing the significantly differentially expressed genes between BGS group and BGS/MCFS group identified by transcriptome sequencing. Cutoff: P value <0.05 and |log2 FC| >1. c) The top 10 biological processes enriched by differentially expressed genes identified by GO analysis. d) The top 10 signal pathways enriched by differentially expressed genes identified by KEGG analysis. e) The chord diagram representing the correlation between the top 10 osteogenic biological processes identified through GO analysis and the top 30 differentially expressed genes associated with osteogenesis ranked with FoldChange. f) t‐distributed Stochastic Neighbor Embedding (t‐SNE) visualizing cell clusters in reduced dimensions. g) The proportion of different subgroup cells in the indicated group. pDCs, plasmacytoid dendritic cells; ECs, endothelial cells; DCs, dendritic cells.

Encouraged by the excellent in vitro and in vivo osteogenesis and angiogenesis properties of BGS/MCFS, transcriptome sequencing was further conducted to explore the corresponding underlying mechanism. The volcano map (**Figure** **6**a) and heatmap (Figure 6b) showed the differentially expressed genes of hBMSCs cultured with BGS/MCFS or BGS, where in all 1410 differentially expressed genes (P < 0.05&|log2 Fold Change| >1) were identified, including 771 activated genes (marked in red) and 639 silenced genes (marked in green). The differentially expressed genes were further enriched in different biological processes by the GO analysis, where 6 in the top 10 enriched biological processes were related to osteogenesis (Figure 6c), suggesting BGS/MCFS can exert a great impact on the osteogenesis process of hBMSCs. Also, the KEGG analysis was conducted and revealed the participation of transforming growth factor‐β (TGF‐β) signal pathway and Wnt signal pathway in the osteogenic and angiogenic processes induced by BGS/MCFS (Figure 6d).

To further identify the specific signal pathway regulating the vascularized osteogenesis of BGS/MCFS, Gene Set Enrichment Analysis (GSEA) was carried out using the GSEA analysis tool, where the TGF‐β signal pathway was found to be significantly activated in BGS/MCFS group compared with BGS group (Figure S28a,b, Supporting Information). More importantly, BMP as ligands for TGF‐β superfamily possessing osteogenic effects were activated (Figure S28c, Supporting Information). The upregulation of a downstream osteoblastgenic aptamer named Runt‐related transcription factor 2 (RUNX2) was also observed (Figure S28d, Supporting Information) in BGS/MCFS group, which suggests the TGF‐β signal pathway stand a good chance of leading the effects of BGS/MCFS‐induced osteogenesis. Furthermore, the top 10 osteogenic biological processes identified by GO analysis and the top 30 differentially expressed genes related to vascularized osteogenesis (ranked by FoldChange) were correlated in a chord diagram for visualization (Figure 6e) of the most engaged genes, where BMP‐6 (marked in red) and endoglin (ENG) were identified as typical osteogenic and angiogenic genes, respectively. Interestingly, apart from BMP‐6 being a potent osteogenic ligand subjecting to the TGF‐β signal pathway, ENG is also one of the TGF‐β receptor complexes as type I transmembrane glycoprotein located on the cellular membrane and regulates the downstream VEGF expression thus participating in angiogenesis.^[^ ^56^ ^]^ Thus, the TGF‐β signal pathway was supposed to significantly contribute to the enhanced vascularized osteogenesis processes of BGS/MCFS.

To elucidate the differentially regulated cell subsets in response to BGS/MCFS or BGS, single‐cell RNA‐sequencing of in situ cell clusters was performed 2 weeks after scaffold implantation. After data filtering, 19015 cells were recognized, with 10147 in the BGS group and 8868 in the BGS/MCFS group. Integrated analysis identified 12 cellular subpopulations, of which a notable increase in the number of osteoblasts (3070 vs 1436) was observed in the BGS/MCFS group compared to BGS. Besides, despite the endothelial cells accounting for a small proportion in identified cell population, significantly more endothelial cells (106 vs 48) were found in the BGS/MCFS group, suggesting the upregulated vascularization in BGS/MCFS group (Figure 6f,g). In addition, significantly more macrophages (3330 vs 2341) were also identified in the BGS/MCFS group, which are considered to contribute to local inflammatory microenvironment response to injury that plays a very important role in initiating early‐phase bone repair (Figure 6f,g).^[^ ^57^ ^]^ Moreover, given the pivotal role of neutrophils in initiating immune responses and facilitating rejection reactions,^[^ ^58^ ^]^ the observed increase in neutrophil count within the BGS group suggests a heightened rejection response, implying a potentially inferior biocompatibility of BGS compared to BGS/MCFS. In summary, single cell sequencing further illustrates the vascularized osteogenic mechanism of BGS/MCFS at cell level.

**Figure 7:**
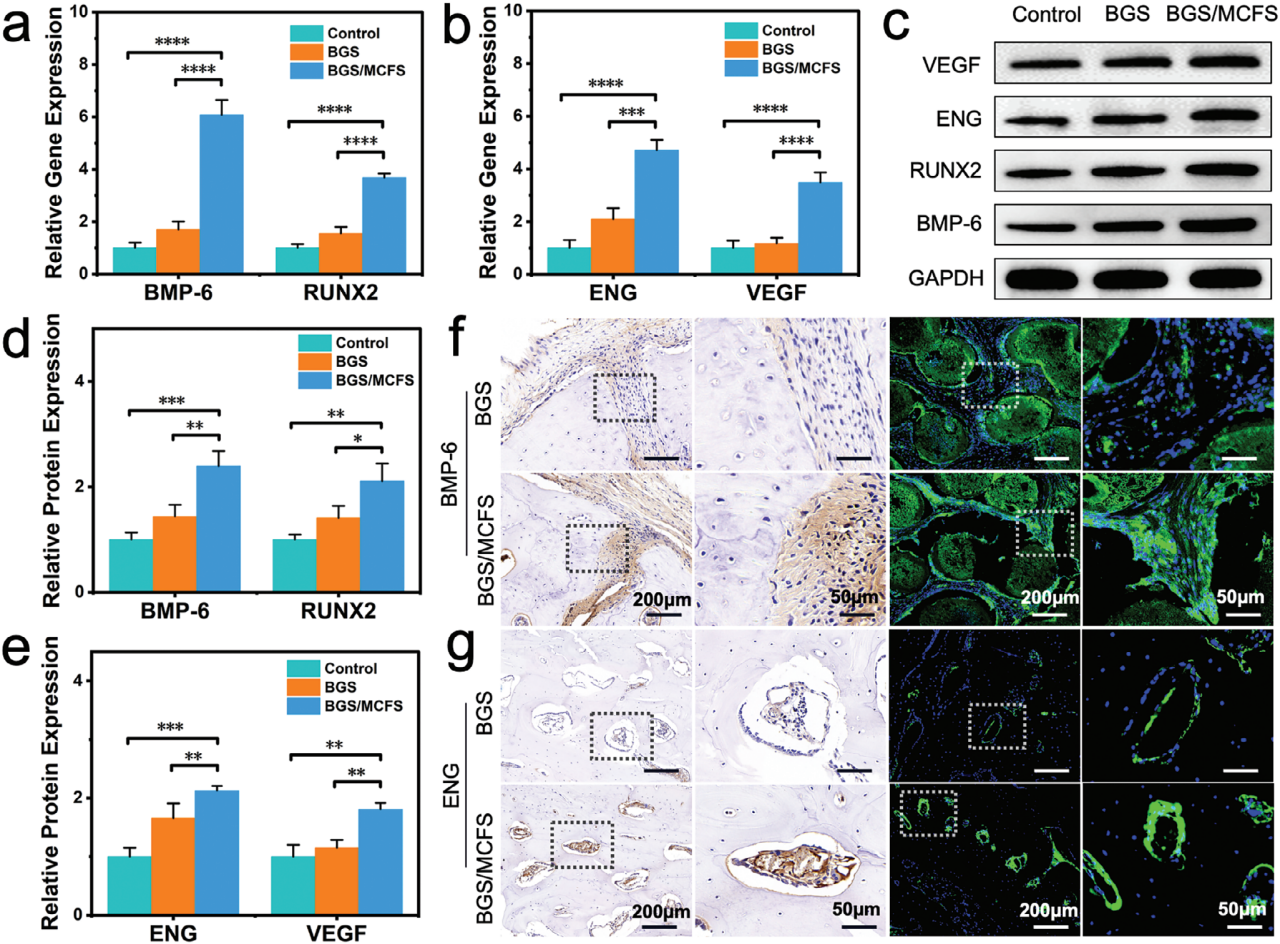
Verification of the vascularized osteogenic mechanism of BGS/MCFS. a) qPCR results determining the expression level of osteogenic genes (BMP‐6, RUNX2) and b) angiogenic genes (ENG, VEGF) of hBMSCs co‐cultured with BGS or BGS/MCFS. c) Western blot detecting the osteogenic and angiogenic proteins (BMP‐6, RUNX2, ENG, and VEGF) expression of hBMSCs co‐cultured with BGS or BGS/MCFS. Quantitative analysis of the d) osteogenic and e) angiogenic proteins expression based on the Western blot bands using ImageJ 1.52v software. Immunohistochemistry and immunofluorescence images detecting the f) BMP‐6 and g) ENG expression around the BGS and BGS/MCFS. Data are expressed as mean ± standard deviation (S.D.) (n = 3). Statistical comparisons were made by one‐way ANOVA (for multiple comparisons): ^*^ *p* < 0.05, ^**^ *p* < 0.01, ^***^ *p* < 0.001, ^****^ *p*  < 0.0001.

Despite transcriptome sequencing results suggesting the TGF‐β signal pathway may account for the osteogenic and angiogenic properties of BGS/MCFS, differentially expressed gene do not necessarily result in changes in downstream protein expression, so further molecular and morphological verifications were performed. First, quantitative polymerase chain reaction (qPCR) assays were carried out to confirm the transcriptional level of the osteogenic genes (BMP‐6 and RUNX2) and angiogenic genes (ENG and VEGF). It turned out the qPCR data were consistent with the transcriptome sequencing results, where the expression level of BMP‐6 and RUNX2 of hBMSCs co‐cultured with BGS/MCFS were 3.6‐fold and 2.4‐fold higher than that with BGS (**Figure** **7**a). Similarly, ENG and VEGF were also upregulated by 2.2‐fold and 3.0‐fold in BGS/MCFS group compared with BGS (Figure 7b). Subsequently, the Western blot assays were performed to evaluate the protein translation level of these osteogenic and angiogenic genes related to TGF‐β signal pathway. As shown in Figure 7c, obviously thicker protein bands of BMP‐6, RUNX2, ENG, and VEGF were observed in BGS/MCFS group compared with control and BGS groups. Further quantitative analysis of the protein bands revealed significantly enhanced fluorescence intensity of BMP‐6, RUNX2, ENG, and VEGF in BGS/MCFS group compared with that in BGS group (Figure 7d,e). In addition, immunohistochemistry and immunofluorescence staining of the decalcified skull sections were also performed for in vivo mechanism verification. As shown in Figure 7f, significantly more BMP‐6 expression was detected around the regenerated bone in BGS/MCFS group compared with BGS group in both immunohistochemistry and immunofluorescence images. Similarly, higher ENG expression revealed by deeper histochemical staining and stronger fluorescence intensity was found in the BGS/MCFS group compared with BGS group (Figure 7g). Taken together, these in vitro and in vivo results verify the outstanding osteogenic and angiogenic properties of BGS/MCFS were mediated by the TGF‐β signal pathway.

## 3 Conclusion

In conclusion, we developed a multifunctional BGS/MCFS with prophylactic/therapeutic capabilities against periprosthetic infections and remarkable vascularized osteogenic properties. The MCFS NSs containing metallic ions demonstrated potent antibacterial efficiency by inhibiting bacterial energy production and nutrient metabolism. More importantly, the excellent NIR‐II photothermal conversion efficiency of MCFS led to a scaffold temperature increase approaching 70 °C under light irradiation, which showed a thorough bacteria elimination ability (with an efficiency of 100%) in a rabbit tibial periprosthetic infection model. The bacterial eradication capabilities highlight the superiority of BGS/MCFS compared to previously reported functionalized BGS. In addition, the incorporation of MCFS into the 3D‐printed BGS resulted in increased surface roughness and 1.7‐fold enhanced cellular adhesion. Sustained released Mg^2+^ and Cu^2+^ from MCFS endowed BGS/MCFS with excellent osteogenic and angiogenic properties, as an 8.5‐fold increase in regenerated bone mass and a 2.3‐fold increase in neovascularization were observed in BGS/MCFS group compared with BGS group in a rabbit critical‐size cranial defect model, which is remarkably preponderant than other previously reported modified BGS. The activation of the TGF‐β signaling pathway was identified as a contributor to the osteogenic and angiogenic properties of BGS/MCFS. Single‐cell sequencing further revealed a significant increase in osteoblasts and endothelial cells around BGS/MCFS, which also accounts for the enhanced vascularized osteogenesis properties of BGS/MCFS. Thus, our study demonstrated that BGS/MCFS capable of preventing/treating periprosthetic infections and promoting vascularized osteogenesis holds significant promise in simultaneously addressing periprosthetic infection and prosthetic loosening.

## Conflict of Interest

The authors declare no conflict of interest.

## Supporting information

Supporting Information

## Data Availability

The data that support the findings of this study are available from the corresponding author upon reasonable request.
