# Supplementary material for: A LDH‐Derived Metal Sulfide Nanosheet‐Functionalized Bioactive Glass Scaffold for Vascularized Osteogenesis and Periprosthetic Infection Prevention/Treatment
Source: Adv Sci (Weinh). 2024 Aug 19;11(39):2403009. doi: 10.1002/advs.202403009 (PMC11497026; doi:10.1002/advs.202403009)
Supplement: Supplementary file 1 — Supporting Information [file ADVS-11-2403009-s001.docx]

***Supporting Information***

**A LDH-Derived Metal Sulfide Nanosheet-Functionalized Bioactive Glass Scaffold for Vascularized Osteogenesis and Periprosthetic Infection Prevention/Treatment**

Yixin Bian, Tingting Hu, Kexin Zhao, Xuejie Cai, Mengyang Li, Chaoliang Tan,* Ruizheng Liang,* Xisheng Weng*

These authors contributed equally: Yixin Bian and Tingting Hu.

Y. Bian, X. Cai and Prof. X. Weng

Department of Orthopedic Surgery, State Key Laboratory of Complex Severe and Rare Diseases, Peking Union Medical College Hospital, Chinese Academy of Medical Science and Peking Union Medical College, Beijing 100730, China

E-mail: drwengxsh@163.com (X. Weng)

T. Hu and Prof. C. Tan

Department Electrical and Electronic Engineering, The University of Hong Kong, Pokfulam Road, Hong Kong SAR 999077, P. R. China.

E-mail: cltan@hku.hk (C. Tan)

K. Zhao, M. Li and Prof. R. Liang

State Key Laboratory of Chemical Resource Engineering, Beijing Advanced Innovation Center for Soft Matter Science and Engineering, Beijing University of Chemical Technology, Beijing 100029, P. R. China.

E-mail: liangrz@mail.buct.edu.cn (R. Liang)

Prof. R. Liang

Quzhou Institute for Innovation in Resource Chemical Engineering, Quzhou 324000, P. R. China.

**Experimental Section**

***Material*:** Cu(NO_3_)_2_·3H_2_O, Mg(NO_3_)_2_·6H_2_O, Fe(NO_3_)_3_·9H_2_O and formamide were acquired from Aladdin Industrial Corporation (Shanghai, China). NaNO_3_, sodium hydroxide (NaOH) and TAA were supported by Macklin Biochemical Co., Ltd (Shanghai, China).

***Fabrication of MgCuFe-LDH precursor*:** MgCuFe-LDH NSs were synthesized using a bottom-up method^45^. In a typical procedure, solution A: Mg(NO_3_)_2_·6H_2_O (0.128 g), Cu(NO_3_)_2_·3H_2_O (0.1206 g) and Fe(NO_3_)_3_·9H_2_O (0.2023 g) were dissolved in deionized (DI) water (50 mL). Solution B: NaNO_3_ (0.0425 g) was dissolved in DI water (50 mL) containing 23% v/v formamide. Solution C: NaOH (0.3 g) was dissolved in DI water (50 mL). Subsequently, solution A and solution C were added dropwise into solution B with magnetic stirring for 0.5 h in N_2_ atmosphere at room temperature. The resulting MgCuFe-LDH NSs were centrifuged and washed by ethanol and water successively at 7000 rpm for 3 min, followed by dialysis (3 kDa) to ensure the excessive formamide was removed.

***Preparation of MCFS NSs*:** The MCFS NSs were synthesized through in situ sulfurization process using MgCuFe-LDH NSs as the precursor. In brief, 0.12 mol TAA was dissolved in 40 mL ethanol, and then 0.024 mol of MgCuFe-LDH NSs were added into the TAA solution, followed by stirring at room temperature for 30 min. After hydrothermal treatment at 120 ℃ for 12 h, the sample was gathered by centrifugation (10000 rpm, 3 min) and rinsed repeatedly with water and ethanol. The final MCFS NSs were re-dispersed in DI water for subsequent experiments.

***Synthesis of BGS/MCFS composite scaffolds*:** The bioactive glass scaffolds (BGS) were placed in of MCFS NSs suspension (1 mg mL^−1^) for 5 min, and then ultrasound treatment was applied for 5 min. Finally, the composite scaffolds were dried thoroughly in a 60 ℃ oven. The whole process was repeated three times.

***Characterization*:** XRD patterns of the samples was recorded by Shimadzu XRD-6000 diffractometer (Cu Kα source, Japan). The FT-IR spectra of the samples in the range of 4000-400 cm^−1^ were measured with a Vector 22 spectrophotometer (Bruker, Germany). UV-vis-NIR absorption spectra at 300-900 nm were recorded by Shimadzu U-3000 spectrophotometer (Japan). The element valence states of the samples were characterized by XPS Escalab 250Xi (Thermo Scientific, USA). Zetasizer UV spectrometer (Malvern Instruments, UK) was used to investigate the Zeta potentials of the samples. The content of elements was detected by ICP-AES (Shimadzu ICPS-7500, Japan).

***Investigation of photothermal performance*:** To evaluate the photothermal performance of the samples, 2 mL of different concentrations of the MCFS NSs were added into transparent quartz vial, followed by 1064 nm laser irradiation with various power densities. Simultaneously, infrared thermal images and temperature changes were recorded every 30 s with a thermal infrared imaging device (Fluke Ti400, USA). The photothermal conversion efficiency (PCE) can be calculated by Equation 1:

η=$\frac{hS\left( T_{max}-T_{surr} \right)-Q_{dis}}{I(1-{10}^{-A})}$

Where h represents the heat transfer coefficient of the MCFS NSs, S is the cross-sectional area of the container, T_max_ stands for the maximum temperature of the suspension, T_Surr_ represents the temperature of the surroundings, Q_dis_ stands for the heat associated with light absorbance by the solvent, I represents the incident laser power, and A is the absorbance of the MCFS NSs at 1064 nm. HS can be calculated by Equation 2-4:

hS=$\frac{mc}{\tau_{s}}$

τ_s_=-$\frac{t}{\ln\theta}$

θ=$\frac{T-T_{surr}}{T_{max}-T_{surr}}$

Where m is the solution mass, c represents the heat capacity of water, T, T_surr_ and T_max_ stand for the solution temperature, ambient temperature and the highest steady-state temperature of the solution, respectively. τ_s_ represents the sample system time constant.

***Release study of metal ions*:** The release profiles of Mg^2+^ and Cu^2+^ from BGS@MCFS were analyzed by ICP-AES. BGS@MCFS was dispersed into 8 mL PBS and incubated at 37 ℃ for 35 days. An aliquot (1 mL) of dispersion was collected at a fixed time point each day.

***In vitro antibacterial assays*:** *MASA*, *E. coli*, and *S. aureus* (10^7^ CFU/mL) were co-incubated with 1 mL of MCFS suspension at varied concentrations (0, 25, 50, 100, and 200 μg mL^−1^) for 2h or further irradiated by 1064 nm laser irradiation (1 W cm^−2^, 10 min). The remaining bacteria were then diluted and inoculated on a prepared LB medium in a humidified incubator with a constant temperature of 37°C for 12 h. Cultivated bacterial colonies were imaged by a digital camera (Fujifilm, Tokyo, Japan) and counted by ImageJ 1.52v software. To access the antibacterial efficiency of BGS/MCFS more intuitively, 10^8^ CFU/mL bacteria were dropped onto the scaffolds and allowed to deposit and adhere for 2h, which was further treated by NIR laser at 0 or 1W cm^−2^ for 10 min. The morphology of irradiated bacteria was observed and imaged under a scanning electron microscope (Zeiss, Jena, Germany) after being dehydrated using gradient ethanol (30%, 50%, 70%, 90%, and 100%).

***RNA sequence for MRSA*:** *MRSA* was cultured with BGS or BGS/MCFS for 6 h and further gathered to extract total RNA using TRIzol reagent (Invitrogen, CA, USA). Subsequently, RNA sequencing was carried out employing TruSeq PE Cluster Kit v3-cBot-HS (Illumina, CA, USA). Differential expression analysis was conducted using the DESeq R package (version 1.18.0). Genes meeting the criteria of |log2FC | > 1 (p-value < 0.05) were identified as differentially expressed genes (DEGs), which were then enriched by the Gene Ontology (GO) analysis and Kyoto Encyclopedia of Genes and Genome (KEGG) analysis.

***In vivo periprosthetic infection treatment*:** A rabbit tibia defect model was made by creating a 5-mm diameter full-thickness circular defect in the upper tibia of New Zealand rabbits. All surgical experiments strictly followed the guidelines of the National Institutes of Health Guidelines for the use of experimental animals, and all experiments were conducted by the guidelines of the Animal Care and Use Committee of Peking Union Medical College Hospital with the ethics number of XHDW-2023-021. Of note, BGS and BGS/MCFS were implanted into the tibia defects after being immersed in the *MRSA* suspension (10^8^ CFU/mL) for 2 h to induce postoperative periprosthetic infection. The bacterial-loaded scaffolds in BGS + NIR-II and BGS/MCFS + NIR-II groups were irradiated by a 1064 nm laser (0 or 1 W cm^−2^, 10 min) through tibial skin 1 day after transplantation. One month later, the tibiae of New Zealand white rabbits were harvested and fixed in paraformaldehyde for Micro-CT scanning (Siemens, Erlangen, Germany). The harvested tibial samples were further decalcified and sliced for H&E staining. The osteogenesis-evaluated H&E staining was also performed on a parallel control series using a rabbit proximal tibial non-infection bone defect model. Muscle tissues (100 mg per rabbit) around the bacterial-adhered scaffolds were also harvested, homogenated, and inoculated on agar medium for following bacterial colony forming observation. The bacterial colonies were imaged by a digital camera (Fujifilm, Tokyo, Japan) and counted by ImageJ 1.52v software.

***In vitro biocompatibility evaluation*:** BGS and BGS/MCFS were sterilized and used for all following cell and animal experiments. CCK-8 assay was conducted to assess the biocompatibility of BGS/MCFS. Specifically, BGS or BGS/MCFS were put into the upper chamber of a 24-well Transwell plate with 3 µm diameter pores (Corning, NY, USA), and the group without any scaffold placed was set as the control group. Ten thousand hBMSCs or HUVECs were then seeded on the lower chamber with medium replaced daily. The cellular proliferation activities were detected on days 1, 3, 5, and 7 using a CCK-8 kit (Sigma, St Louis, MO, USA) at the OD values 450 ± 5 nm in a multifunctional full-wavelength microplate reader (Varioskan Flash, Thermo Fisher Scientific, USA).

***Cell adhesion assay*:** Ten thousand hBMSCs were seeded on BGS or BGS/MCFS in 96-well plates and allowed for adhesion for 24h. Then the cell-seeded scaffolds were fixed with paraformaldehyde and glutaraldehyde and stained with DAPI and rhodamine-phalloidin (Sigma, St Louis, MO, USA) for 30 min, which were further transferred into CLSM-exclusive culture dish and scanned and reconstructed *via* CLSM (Leica, Wetzlar, Germany). Alternatively, the cell-seeded scaffolds were further subjected to dehydration using gradient ethanol (30%, 50%, 70%, 90%, and 100%) for detailed morphological observation of hBMSCs under a scanning electron microscope (Zeiss, Jena, Germany).

***In vitro osteogenic properties evaluation*:** Initially, 20,000 hBMSCs were seeded on the lower chamber of a 24-well Transwell plate with 3 µm diameter pores (Corning, NY, USA) for 24 h allowing cell adhesion and proliferation. Then the BGS or BGS/MCFS was put into the upper chamber of the Transwell plate to induce osteogenic differentiation of hBMSCs, while the control group put nothing on the upper chamber and only underwent daily medium replacement. Seven and fourteen days post-co-culture, hBMSCs were fixed with paraformaldehyde and subjected to staining for ALP using the Alkaline Phosphatase Detection Kit (Sigma-Aldrich, St Louis, USA) or alizarin red S using the Alizarin Red S Kit (Solaibao, Beijing, China), respectively. Subsequently, stained cells were imaged using a digital camera (Fujifilm, Tokyo, Japan) and an optimal inverted microscopy (Olympus, Tokyo, Japan) to assess ALP expression or calcium deposition levels. Randomly selected microscopic fields of staining were quantitatively analysed using ImageJ 1.52v software.

***Scratch assay*:** HUVECs were seeded on the lower chamber of a 6-well Transwell plate with 3 µm diameter pores (Corning, NY, USA) until cellular contact inhibition was observed. After the scratches were created with a 200-μL pipette tip, BGS or BGS/MCFS were put into the upper chamber of the Transwell plate while the control group did not put any scaffold. Randomly selected cell migration images were captured using optimal inverted microscopy (Olympus, Tokyo, Japan) at 0 h and 24 h after co-culture. Further calculations were made using the formula below for HUVEC migration ratios as migration rate (%) = (A*_0_*–A*_n_*)/A*_0_*×100%, where A*_0_* stands for the initial scratch area and A*_n_* stands for the remaining scratch area 24 h after co-culture.

***Transwell assay*:** Ten thousand HUVECs were seeded on the upper chamber of 24-well Transwell plates (8-μm pore filters, Corning, NY, USA) and incubated by a serum-free medium. Meanwhile, BGS or BGS/MCFS were placed in the lower chamber of Transwell plates while nothing was placed in the lower chamber in the control group. After 24 h of co-incubation, the HUVECs migrated to the lower layer and were fluorescently stained with Calcein-AM (Sigma, St Louis, MO, USA) and imaged by an inverted fluorescence microscope (Olympus, Tokyo, Japan).

***Tube formation assay*:** HUVECs were seeded on Matrigel (Sigma, St Louis, MO, USA) in the lower chamber of a 24-well Transwell plate (3-μm pore filters, Corning, NY, USA) at a density of 20000/well and BGS or BGS/MCFS were placed in the upper chamber of Transwell plates while nothing was placed in the upper chamber in the control group. After incubation for 6 h, the tube formation capabilities of BGS and BGS/MCFS were evaluated by optimal inverted microscopy (Olympus, Tokyo, Japan). Further quantitative analysis of the joint number and the total tube length was performed with ImageJ 1.52v software.

***In vivo osteogenesis and angiogenesis evaluation*:** Critical-sized calvaria defect models of New Zealand White Rabbits were used to access the osteogenic capabilities of BGS and BGS/MCFS. Notably, two 5-mm diameter full-thickness circular defects were made in the skull of New Zealand White Rabbits and were subsequently filled by BGS and BGS/MCFS. Fluorescent dyes including Calcein-AM and Alizarin red S (Sigma, St Louis, MO, USA) were intraperitoneally injected 4 and 6 weeks after scaffold implantation to mark regenerated bone tissue. Four and eight weeks after the operation, the rabbits were anesthetized followed by injecting MICROFIL® (Flow Tech, Carver, MA, USA) from the heart to fulfill the entire microvascular network. The vascularized skulls were then harvested for Micro-CT scanning and 3D reconstruction (Siemens, Erlangen, Germany) to evaluate and analyze regenerated bone and vascular tissues. Furthermore, the skulls were decalcified and sectioned for H&E staining and toluidine blue staining. Meanwhile, the fluorescence-labeled regenerated bone tissues were imaged *via* CLSM (Leica, Wetzlar, Germany).

***Transcriptome sequencing*:** The BGS and BGS/MCFS were put in the upper chamber of a 6-well Transwell plate with 3 µm diameter pores (Corning, NY, USA) while 100000 hBMSCs were seeded on the lower chamber. Seven days after co-culture, the cells were digested and washed with PBS for three times, followed by lysed by 1 mL of TRIzol (Life Technologies Corporation, Carlsbad, USA). RNA extraction kit and DNase (Thermo Fisher, USA) were used to extract RNA from the samples and digest DNA. Subsequently, the extracted RNA was enriched by magnetic beads with Oligo (dT) and interrupted by specialized reagents, which served as templates in the following steps. Single-strand cDNA and double-strand cDNA were synthesized by six-base random primers and a two-strand reaction system and amplified by the selected fragment size. The Agilent 2100 Bioanalyzer was involved to evaluate the constructed library and the Illumina sequencer was utilized for sequencing. R Foundation for Statistical Computing (version 4.2.2; Vienna, Austria) was used for data analyses and image production.

***Single-cell RNA-sequencing analysis*:** Critical-sized calvaria defect model of New Zealand White Rabbits implanted with BGS and BGS/MCFS were sacrificed two weeks after scaffold implantation. Regenerated tissues within the calvarial defect area were collected and washed multiple times in cold PBS, which were then enzymatically digested at 37 °C for 40 min. A 70 μm nylon mesh was employed to filter the resulting mixture for single-cell isolation. Then, mRNA libraries were prepared and sequenced. Cells with over 300 detectable genes, a minimum of 500 read counts, and less than 20% mitochondrial gene expression were retained, which was processed by log-normalization and scaling following the Seurat pipeline. t-distributed Stochastic Neighbor Embedding (t-SNE) was utilized to visualize cellular diversity in reduced dimensions.

***qRT-PCR analysis*:** The method of RNA extraction was the same as that in the section on *Transcriptome Sequencing*. A NanoDrop spectrophotometer (Thermo Fisher Scientific, USA) was utilized to measure the concentration of extracted RNA. Afterward, the RNA was transcribed into cDNA using an RNA-to-cDNA kit (Applied Biosystems, Foster City, USA). Then, RT-PCR was performed on the ABI Step One Plus real-time PCR system (Applied Biosystems, USA) and SYBR Green RT-PCR kit (Takara, Japan) for target gene detection. GAPDH was used as the reference gene and the *∆∆cT* method was involved to determine relative gene expression.

***Western blot assay*:** The co-culture paradigm of scaffold and hBMSCs was the same as that in the section on *Transcriptome Sequencing*. RIPA Lysis and Extraction Buffer (Invitrogen, Carlsbad, CA) was used to extract the total proteins and PierceTM Rapid Gold BCA Protein Assay Kit (Invitrogen, Carlsbad, CA) was employed to quantify the protein concentration. The extracted proteins were subjected to a polyvinylidene difluoride (PVDF) membrane and blocked by 5% dilute skim milk. After washing with Tris Buffered Taline Tween (TBST), the protein-loaded PVDF membrane was treated with primary antibody at 4 °C overnight and second antibody at room temperature for 30 min. Enhanced chemiluminescence (ECL, Thermo Fisher Scientific, CA, USA) was utilized to visualize the target bands and Bio-Rad image analysis software (Bio-Rad, Hercules, CA, USA) was utilized for further quantitative analysis.

***Immunohistochemistry and immunofluorescence*:** Proteinase K (Sigma-Aldrich) and 3% H_2_O_2_ were used to digest and eliminate endogenous peroxidase of the skull slide. The slides were incubated with the primary antibody at 4 °C overnight after being blocked by goat serum, and further incubated with the second antibody at room temperature for 30 min. The histochemistry images were taken by optimal inverted microscopy (Olympus, Tokyo, Japan) and the fluorescent images were captured *via* CLSM (Leica, Wetzlar, Germany).

**Statistical analysis and clarification.** Data are expressed as mean ± standard deviation (S.D.). Statistical comparisons were made by one-way ANOVA (for multiple comparisons): *p < 0.05, **p < 0.01, *** p < 0.001, **** p < 0.0001. The n-values in *in vitro* bacterial and cellular assays represent technical repetition, while the n-values in *in vivo* antibacterial and vascularized osteogenesis assays represent biological repetition.

**Table S1.** Comparison of the antibacterial efficiency of functionalized BGS reported in previous studies.

| **Modification Strategies** | **Efficiency for drug-resistance bacteria** | **Efficiency for gram-positive bacteria** | **Efficiency for gram-negative bacteria** | **Co-incubation time (hours)** | **Ref.** |
| --- | --- | --- | --- | --- | --- |
| **MCFS** | **100% (*MRSA*)**  **(with NIR-II)** | **100%**  **(*S. aureus*) (with NIR-II)** | **100%**  **(*E. coli*)**  **(with NIR-II)** | **2** | **This work** |
| Silver | ~58% (*MRSA*) | / | / | 24 | [1] |
| Copper | / | ~60%  (*S. aureus*) | ~77%  (*E. coli*) | 72 | [2] |
| Polydopamine | / | ~99%  (*S. aureus*) | ~99%  (*E. coli*) | 6 | [3] |
| Cerium | / | ~99%  (*S. aureus*) | ~60%  (*E. coli*) | 24 | [4] |
| Single atomic iron catalyst | / | 100%  (*S. aureus*)  (with NIR-I) | 100%  (*E. coli*)  (with NIR-I) | 4 | [5] |
| Amine-functionalized copper and epidermal growth factor | / | ~ 70%  (*E. faecalis*) | / | 5 | [6] |
| Mixed-valence molybdenum | ~99%  (*MRSA*) | ~99%  (*S. aureus*) | ~99%  (*E. coli*) | 12 | [7] |
| Tellurium | / | ~95%  (*S. aureus*) | ~95%  (*E. coli*) | 48 | [8] |
| Gallium | / | ~99%  (*S. aureus*) | ~99%  (*E. coli*) | 24 | [9] |
| Copper /alendronate sodium/Pluronic^®^F127-CHO | ~99%  (*MRSA*) | ~95%  (*S. aureus*), | ~95%  (*E. coli*) | 3 | [10] |

**Table S2.** Comparison of the osteogenic efficiency of functionalized BGS reported in previous studies.

| **Modification strategy** | **Bone volume (folds)** | **Bone mineral density (folds)** | **Bone mass (folds)** | **Duration (weeks)** | **Ref.** |
| --- | --- | --- | --- | --- | --- |
| **MCFS** | **~3.6** | **~2.4** | **~8.5** | **8** | **This work** |
| Black phosphorus | ~1.8 | ~1.3 | ~2.3 | 8 | [11] |
| CuFeSe_2_ nanocrystals | ~0.9 | ~0.8 | ~0.7 | 8 | [12] |
| Copper | ~1.0 | / | / | 12 | [13] |
| Ti_3_C_2_ MXene | ~1.3 | ~1.2 | ~1.6 | 24 | [14] |
| Nb_2_C MXene wrapped with S-nitrosothiol-grafted mesoporous silica | ~2.0 | ~1.5 | ~3.0 | 16 | [15] |
| Single atomic iron catalyst | ~1.5 | ~1.8 | ~2.7 | 8 | [5] |
| Nb_2_C MXene | ~1.5 | ~1.2 | ~1.8 | 24 | [16] |
| Cerium oxide nanoparticles | ~1.7 | / | / | 12 | [17] |
| Carbon dots-doped bioglass nanoparticles and parathyroid hormone derivative | ~1.5 | ~1.9 | ~2.9 | 8 | [18] |
| Selenium | ~1.4 | ~1.1 | ~1.5 | 8 | [19] |

**Figure S1.** XRD spectra of a) MgCuFe-LDH and b) MCFS NSs.


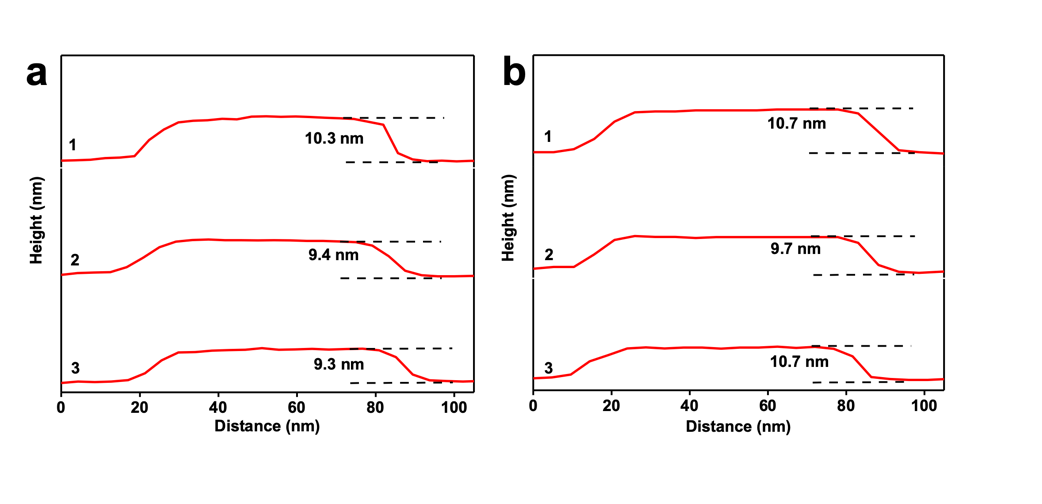


**Figure S2.** The thickness of a) MgCuFe-LDH and b) MCFS NSs measured by AFM.


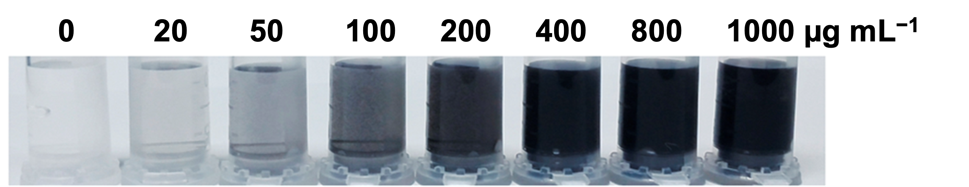


**Figure S3.** Digital photos of MCFS NSs at different concentrations.


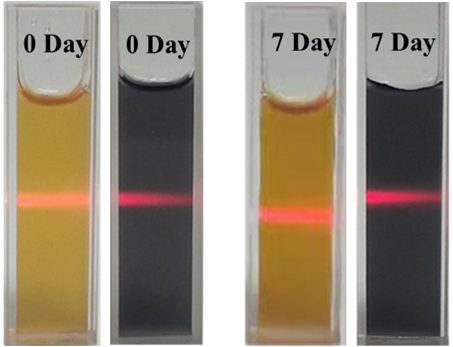


**Figure S4.** The Tyndall effect of MgCuFe-LDH and MCFS NSs in water from day 0 and day 7.


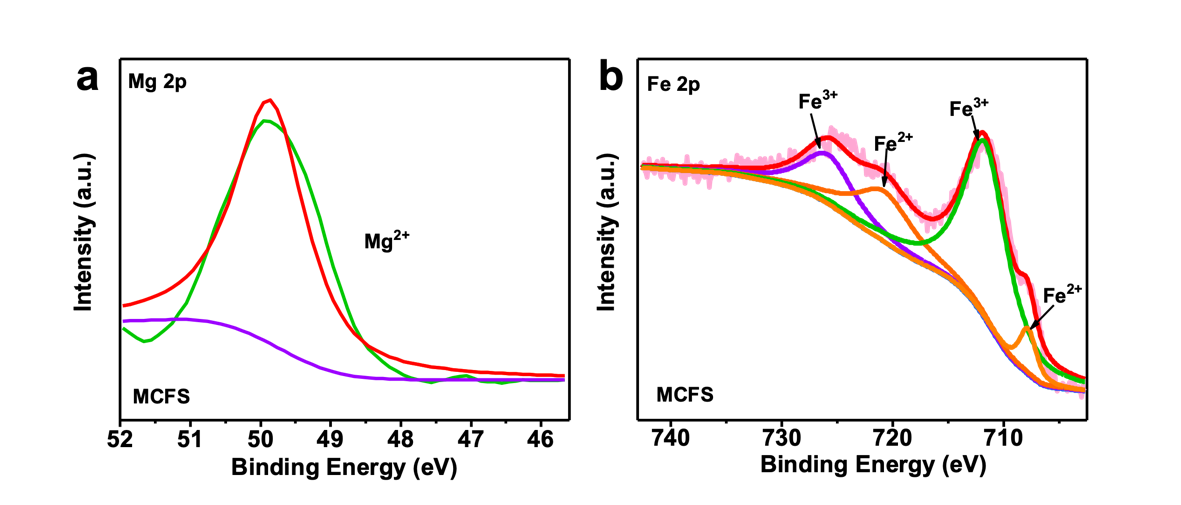


**Figure S5.** a) Mg 2p and b) Fe 2p XPS spectra of MCFS NSs.


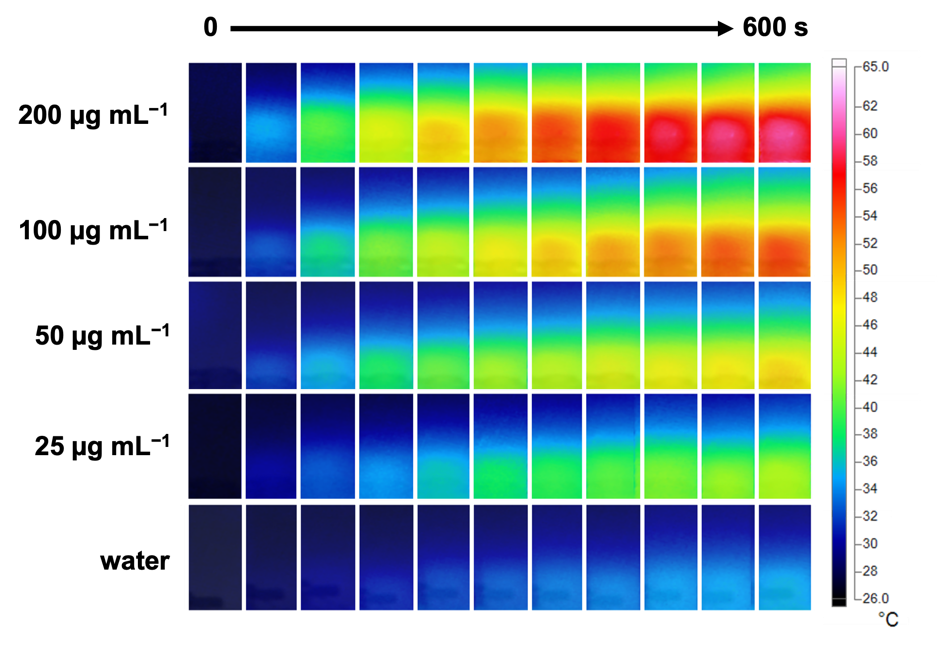


**Figure S6.** Photothermal images of water and MCFS NSs upon 1064 nm irradiation at 1.0 W cm^−2^.


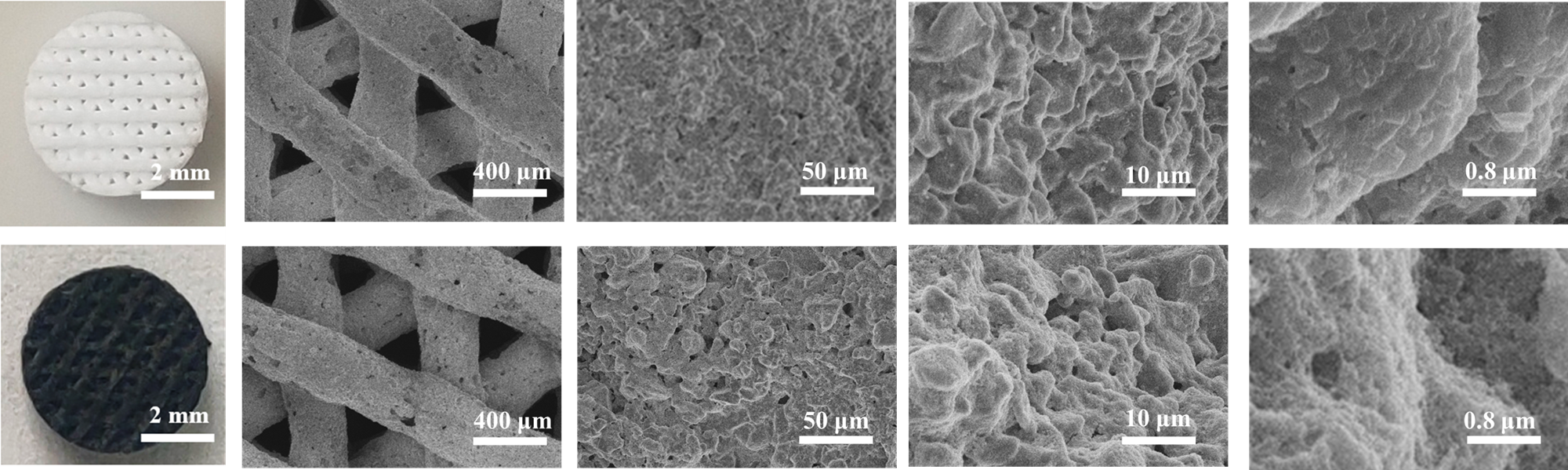


**Figure S7.** Digital photos and SEM images of BGS and BGS/MCFS scaffolds.


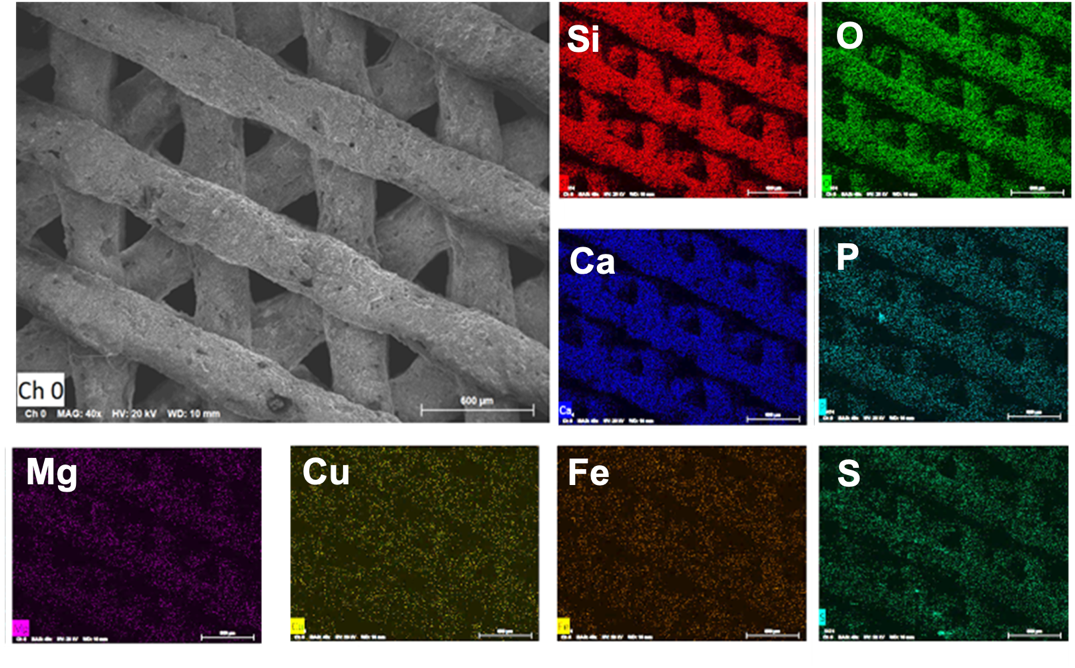


**Figure S8.** Energy dispersive X-Ray spectroscopy (EDX) elemental mapping of BGS/MCFS scaffold.


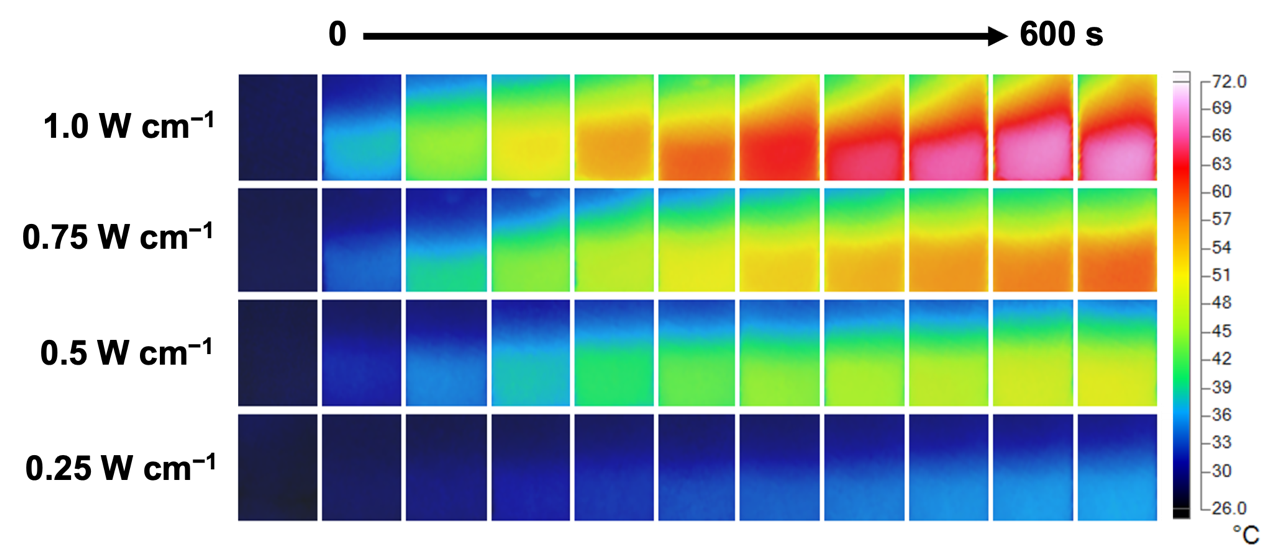


**Figure S9.** Photothermal images of BGS/MCFS scaffolds upon 1064 nm irradiation with different laser power densities (0.25, 0.5, 0.75 and 1 W cm^−2^).


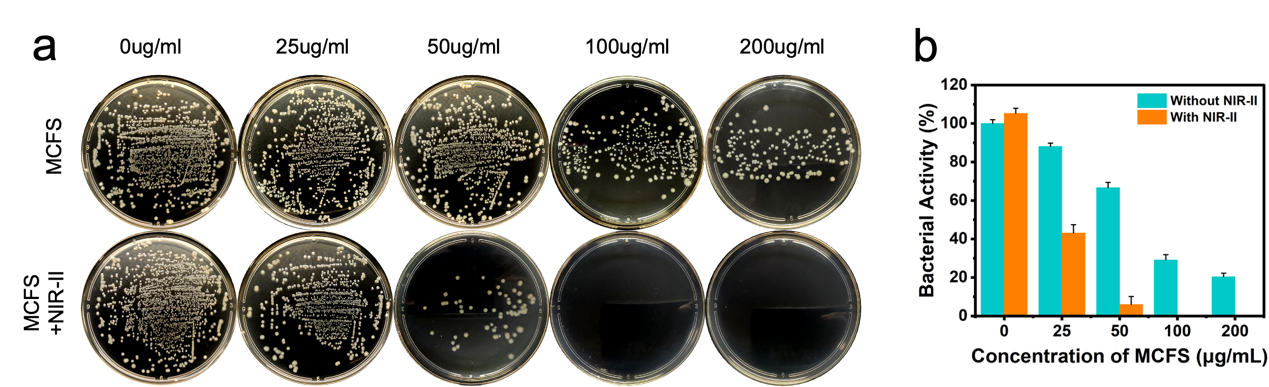


**Figure S10.** a) Digital photographs and b) quantitative profile of the bacterial colony formed by remaining *Escherichia coli* after incubation with different concentration MgCuFe-LDH suspension with or without 1064 nm NIR-II irradiation. Data are expressed as mean ± standard deviation (S.D) (n=3).


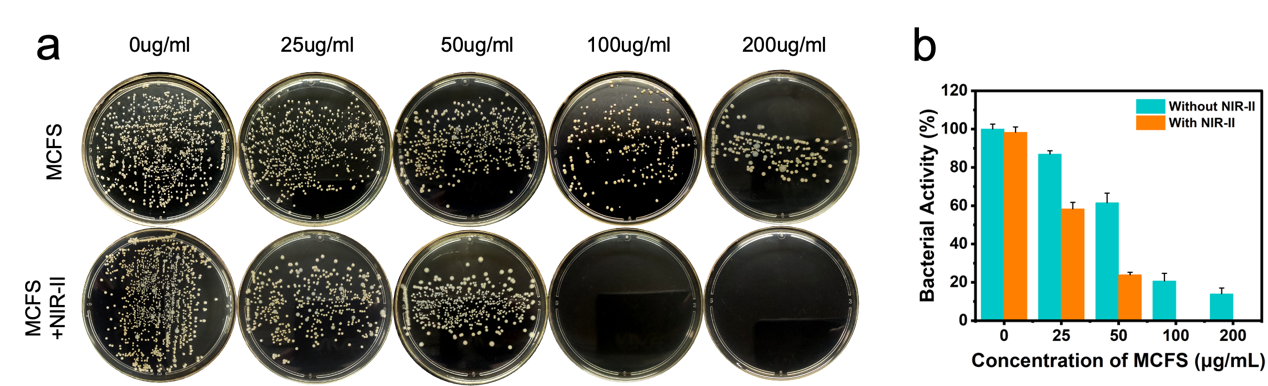


**Figure S11.** a) Digital photographs and b) quantitative profile of the bacterial colony formed by remaining *Staphylococcus aureus* after incubation with different concentration MgCuFe-LDH suspension with or without 1064 nm NIR-II irradiation. Data are expressed as mean ± standard deviation (S.D) (n=3).


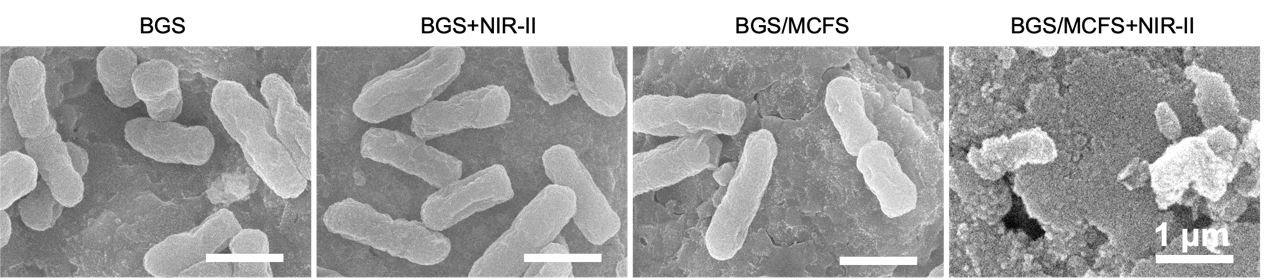


**Figure S12.** SEM images of *Escherichia coli* inoculated on BGS or BGS/MCFS with or without 1064 nm NIR-II irradiation.


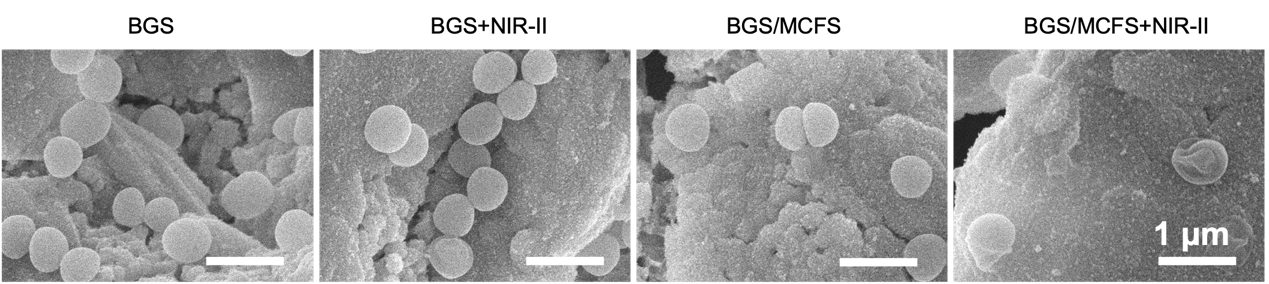


**Figure S13.** SEM images of *Staphylococcus aureus* inoculated on BGS or BGS/MCFS with or without 1064 nm NIR-II irradiation.


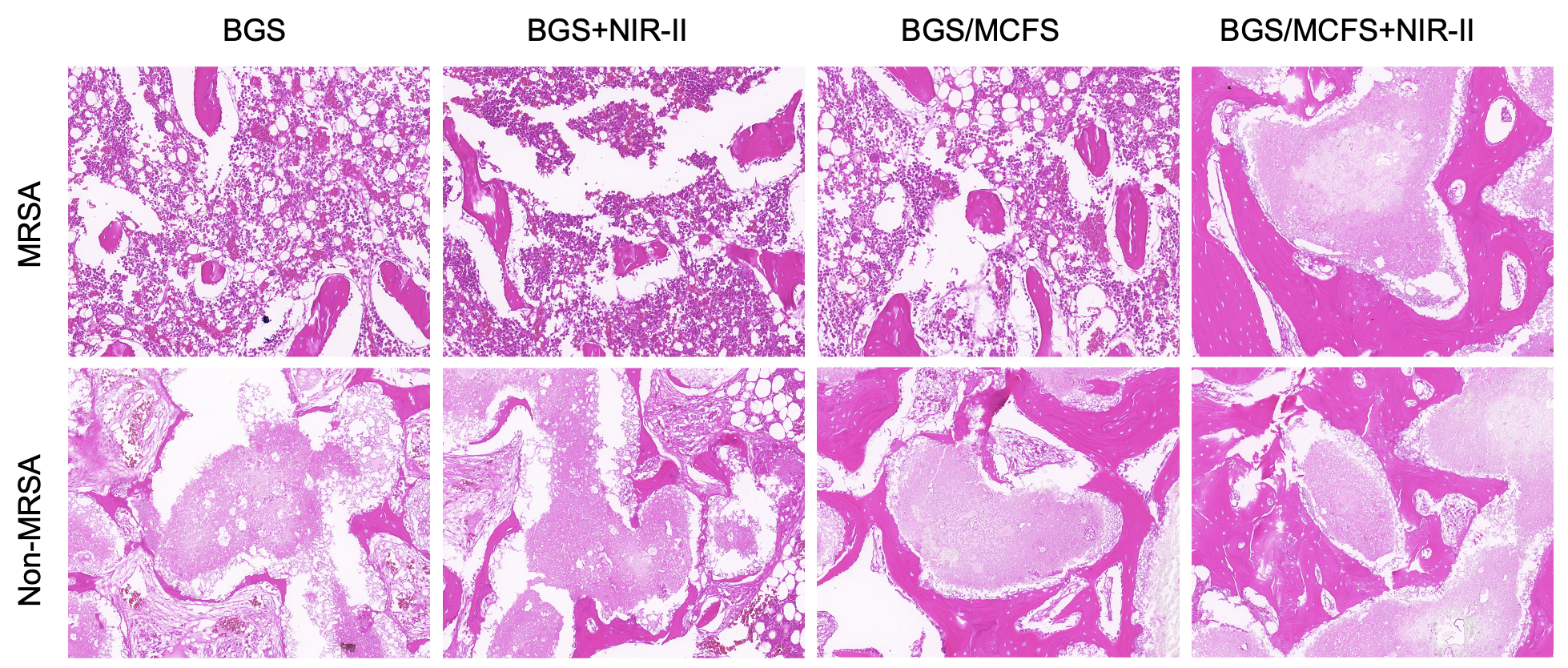


**Figure S14.** H&E staining of regenerated bone in BGS, BGS + NIR-II, BGS/MCFS, BGS/MCFS + NIR-II groups implanted with *MRSA*-adhered (top row) or sterilized scaffolds (bottom row).


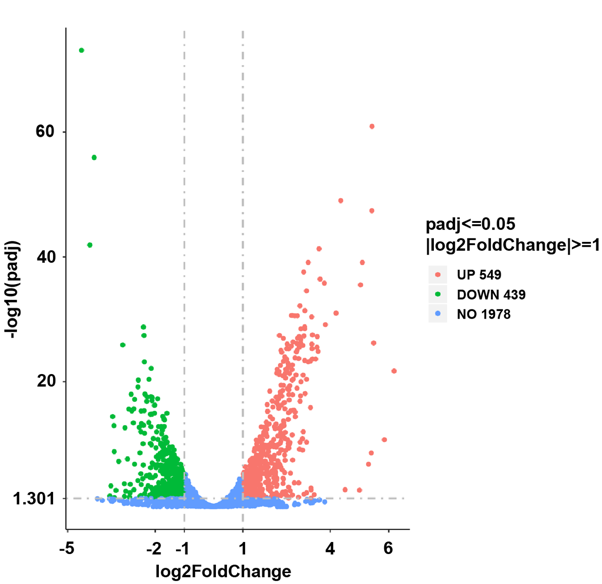


**Figure S15.** The volcano map illustrating the differentially expressed genes of *MRSA* between control group and MCFS NSs group (P < 0.05&|log2 Fold Change| > 1).

***
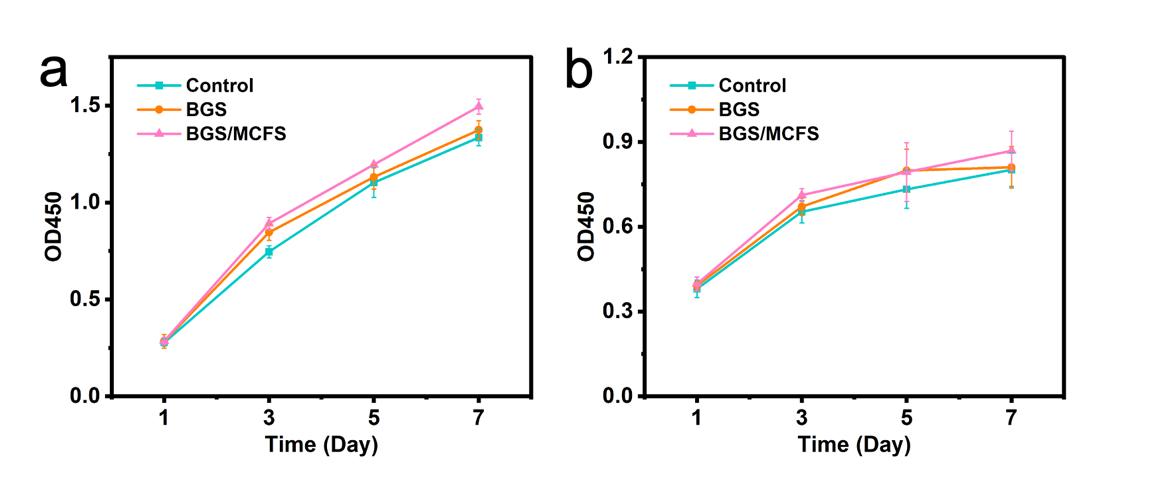
***

**Figure S16.** CCK-8 experiments evaluating the biocompatibility of BGS and BGS/MCFS for a) hBMSCs and b) HUVECs up to the 7th day.


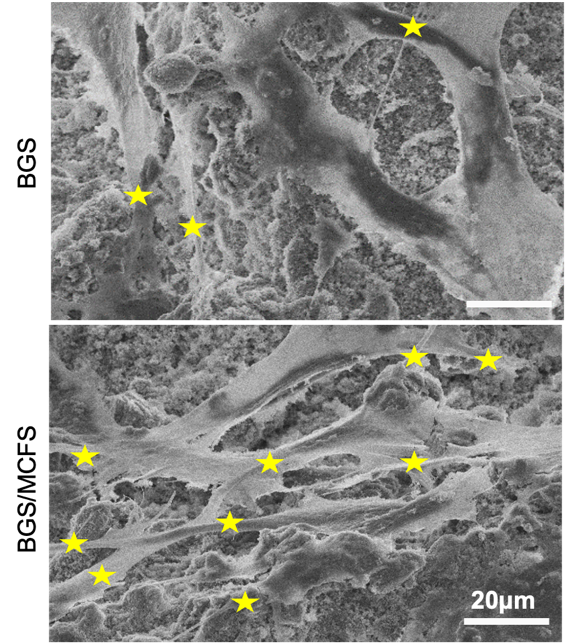


**Figure S17.** SEM images indicating the detailed morphology of hBMSCs adhered on the surface of BGS and BGS/MCFS.


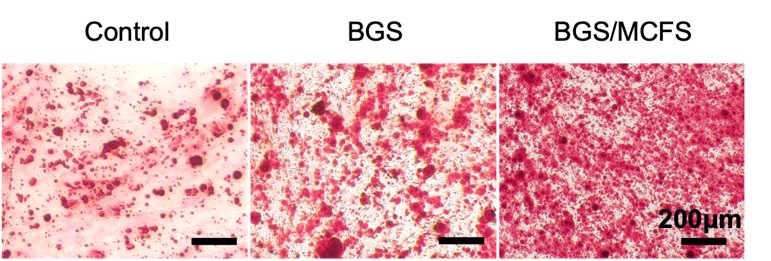


**Figure S18.** Optical microscopic images of alizarin red S staining of hBMSCs in control, BGS, and BGS/MCFS group.


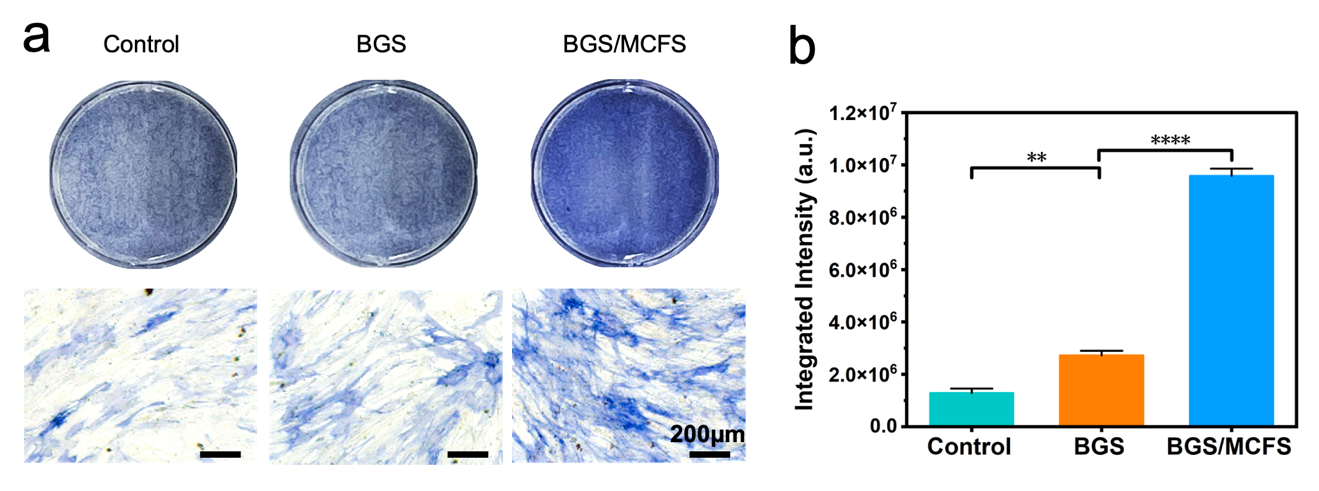


**Figure S19.** a) Digital and optical microscopic images of alkaline phosphatase staining of hBMSCs in control, BGS, and BGS/MCFS group. b) Quantitative analysis of optical microscopic images of alkaline phosphatase staining using ImageJ 1.52v software. Data are expressed as mean ± standard deviation (S.D.) (n=3). Statistical comparisons were made by one-way ANOVA (for multiple comparisons): **p < 0.01, **** p < 0.0001.


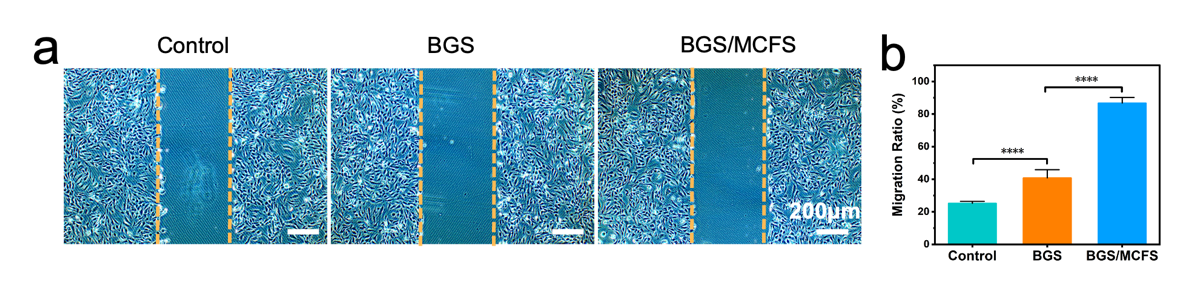


**Figure S20.** a) Optical microscopic images of scratch assay at 0 h in control, BGS, and BGS/MCFS group. b) Quantitative analysis of the migration ratio based on optical microscopic images of scratch assay using ImageJ 1.52v software. Data are expressed as mean ± standard deviation (S.D) (n=3). Statistical comparisons were made by one-way ANOVA (for multiple comparisons): **** p < 0.0001.


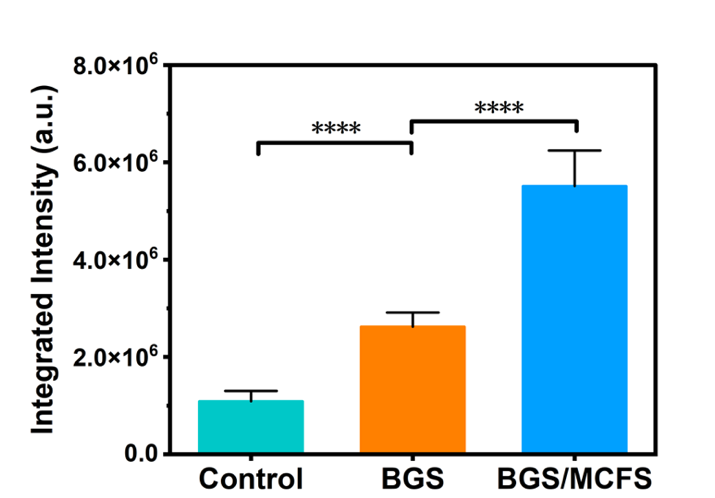


**Figure S21.** Quantitative analysis of the fluorescence intensity of migrated HUVECs in Transwell assay. Data are expressed as mean ± standard deviation (S.D) (n=3). Statistical comparisons were made by one-way ANOVA (for multiple comparisons): **** p < 0.0001.


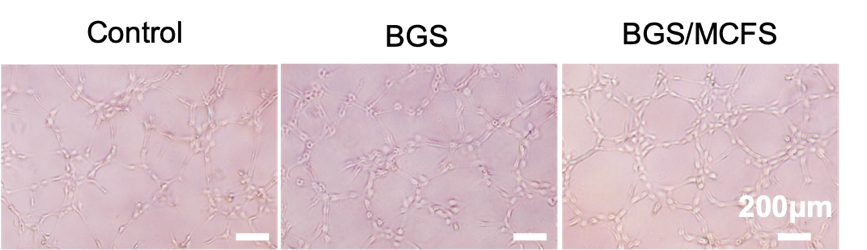


**Figure S22.** Original images of tube formation assay in control, BGS, and BGS/MCFS group.


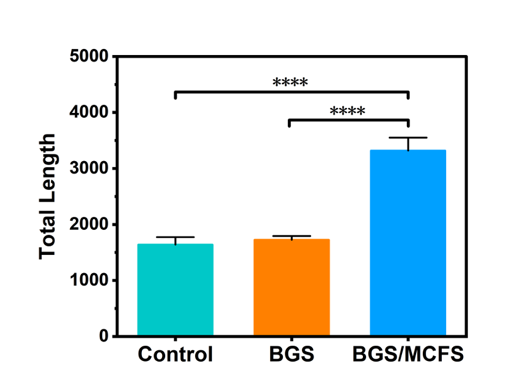


**Figure S23.** Quantitative analysis of the total tube length in tube formation experiments using ImageJ 1.52v software. Data are expressed as mean ± standard deviation (S.D) (n=3). Statistical comparisons were made by one-way ANOVA (for multiple comparisons): **** p < 0.0001.


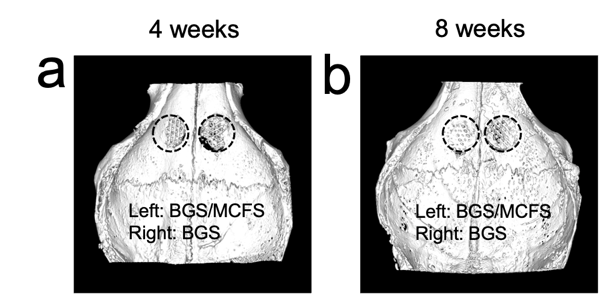


**Figure S24.** Back view of 3D reconstructed Micro-CT images indicating bone regeneration induced by BGS or BGS/MCFS a) 4 and b) 8 weeks after scaffold implantation.


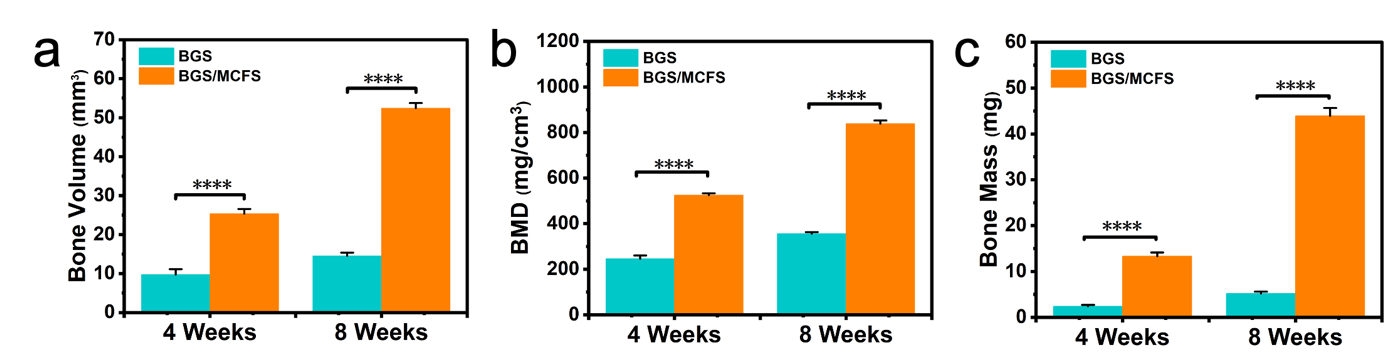


**Figure S25.** Quantitative analysis of the a) volume, b) mineral density, and c) mass of newly-formed bone tissue based on the Micro-CT images. Data are expressed as mean ± standard deviation (S.D) (n=3). Statistical comparisons were made by one-way ANOVA (for multiple comparisons): **** p < 0.0001.


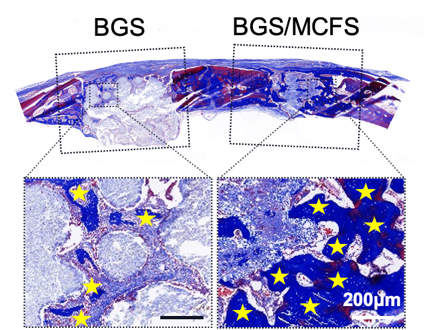


**Figure S26.** Toluidine blue staining of regenerated bone induced by BGS or BGS/MCFS.


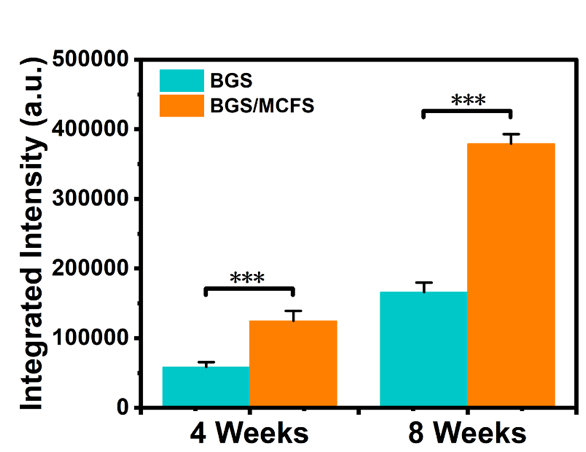


**Figure S27.** Quantitative analysis of the regenerated vessels around BGS or BGS/MCFS. Data are expressed as mean ± standard deviation (S.D) (n=3). Statistical comparisons were made by one-way ANOVA (for multiple comparisons): *** p < 0.001.


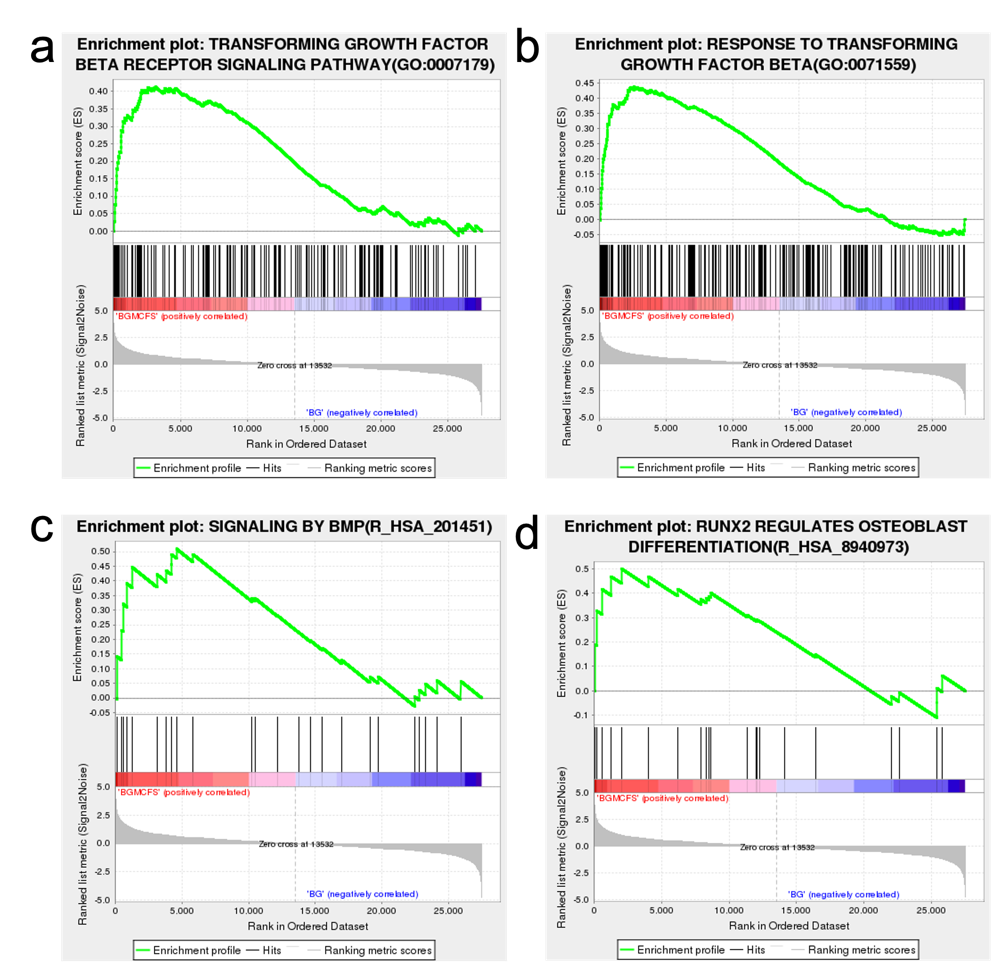


**Figure S28.** a, b) Transforming growth factor-beta, c) bone morphogenetic protein (BMP), d) and Runt-related transcription factor 2 (RUNX2) signal pathways were found to be significantly activated in BGS/MCFS group compared with BGS group revealed by Gene Set Enrichment Analysis.

**References**

[1] A. C. Marsh, N. P. Mellott, N. Pajares-Chamorro, M. Crimp, A. Wren, N. D. Hammer, X. Chatzistavrou, *Bioact. Mater.* **2019**, *4*, 15.

[2] L. R. Rivera, A. Cochis, S. Biser, E. Canciani, S. Ferraris, L. Rimondini, A. R. Boccaccini, *Bioact. Mater.* **2021**, *6*, 1479.

[3] Y. Xue, W. Niu, M. Wang, M. Chen, Y. Guo, B. Lei, *ACS Nano* **2020**, *14*, 442.

[4] F. Kurtuldu, H. Kaňková, A. M. Beltrán, L. Liverani, D. Galusek, A. R. Boccaccini, *Mater. Today Bio.* **2021**, *12*, 100150.

[5] L. Wang, Q. Yang, M. Huo, D. Lu, Y. Gao, Y. Chen, H. Xu, *Adv. Mater.* **2021**, *33*, e2100150.

[6] A. El-Fiqi, N. Mandakhbayar, S. B. Jo, J. C. Knowles, J. H. Lee, H. W. Kim, *Bioact. Mater.* **2021**, *6*, 123.

[7] W. Niu, M. Chen, Y. Guo, M. Wang, M. Luo, W. Cheng, Y. Wang, B. Lei, *ACS Nano* **2021**, *15*, 14323.

[8] Y. Zhang, M. Hu, W. Zhang, X. Zhang, *J. Colloid Interface Sci.* **2022**, *610*, 719.

[9] B. W. Stuart, G. E. Stan, A. C. Popa, M. J. Carrington, I. Zgura, M. Necsulescu, D. M. Grant, *Bioact. Mater.* **2022**, *8*, 325.

[10] L. Zhang, W. Niu, Y. Lin, J. Ma, T. Leng, W. Cheng, Y. Wang, M. Wang, J. Ning, S. Yang, B. Lei, *J. Nanobiotechnology* **2023**, *21*, 162.

[11] B. Yang, J. Yin, Y. Chen, S. Pan, H. Yao, Y. Gao, J. Shi, *Adv. Mater.* **2018**, *30*, 1705611.

[12] W. Dang, T. Li, B. Li, H. Ma, D. Zhai, X. Wang, J. Chang, Y. Xiao, J. Wang, C. Wu, *Biomaterials* **2018**, *160*, 92.

[13] R. Lin, C. Deng, X. Li, Y. Liu, M. Zhang, C. Qin, Q. Yao, L. Wang, C. Wu, *Theranostics* **2019**, *9*, 6300.

[14] S. Pan, J. Yin, L. Yu, C. Zhang, Y. Zhu, Y. Gao, Y. Chen, *Adv. Sci.* **2020**, *7*, 1901511.

[15] Q. Yang, H. Yin, T. Xu, D. Zhu, J. Yin, Y. Chen, X. Yu, J. Gao, C. Zhang, Y. Chen, Y. Gao, *Small* **2020**, *16*, 1906814.

[16] J. Yin, S. Pan, X. Guo, Y. Gao, D. Zhu, Q. Yang, J. Gao, C. Zhang, Y. Chen, *Nanomicro. Lett.* **2021**, *13*, 30.

[17] M. Zhang, X. Zhai, T. Ma, Y. Huang, M. Jin, H. Yang, H. Fu, S. Zhang, T. Sun, X. Jin, Y. Du, C. H. Yan, *ACS Nano* **2023**, *17*, 4433.

[18] S. Liu, Z. Han, J. N. Hao, D. Zhang, X. Li, Y. Cao, J. Huang, Y. Li, *Bioact. Mater.* **2023**, *26*, 1.

[19] D. Chen, Z. Liang, Z. Su, J. Huang, Y. Pi, Y. Ouyang, T. Luo, L. Guo, *ACS Appl. Mater. Interfaces* **2023**, *15*, 34378.
